# Endoplasmic reticulum stress drives proteinuria-induced kidney lesions via Lipocalin 2

**DOI:** 10.1038/ncomms10330

**Published:** 2016-01-20

**Authors:** Khalil El Karoui, Amandine Viau, Olivier Dellis, Alessia Bagattin, Clément Nguyen, William Baron, Martine Burtin, Mélanie Broueilh, Laurence Heidet, Géraldine Mollet, Anne Druilhe, Corinne Antignac, Bertrand Knebelmann, Gérard Friedlander, Frank Bienaimé, Morgan Gallazzini, Fabiola Terzi

**Affiliations:** 1 Mechanisms and Therapeutic Strategies of Chronic Kidney Disease, INSERM U1151—CNRS UMR 8253, Université Paris Descartes, Institut Necker Enfants Malades, Département « Croissance et Signalisation », Hôpital Necker Enfants Malades, 149 Rue de Sèvres, Paris 75015, France; 2 Service d'Explorations Fonctionnelles, Assistance Publique-Hôpitaux de Paris, Hôpital Necker Enfants Malades, 149 Rue de Sèvres, Paris 75015, France; 3 UMR-S 757 INSERM, Université Paris Sud 11, Rue des Adèles, Orsay 91405, France; 4 INSERM U1016, CNRS UMR 8104, Université Paris Descartes, Institut Cochin, Paris, France; 5 INSERM U1163, Université Paris Descartes, Institut Imagine, Hôpital Necker Enfants Malades, 149 Rue de Sèvres, Paris 75015, France

## Abstract

In chronic kidney disease (CKD), proteinuria results in severe tubulointerstitial lesions, which ultimately lead to end-stage renal disease. Here we identify 4-phenylbutyric acid (PBA), a chemical chaperone already used in humans, as a novel therapeutic strategy capable to counteract the toxic effect of proteinuria. Mechanistically, we show that albumin induces tubular unfolded protein response via cytosolic calcium rise, which leads to tubular apoptosis by Lipocalin 2 (LCN2) modulation through ATF4. Consistent with the key role of LCN2 in CKD progression, *Lcn2* gene inactivation decreases ER stress-induced apoptosis, tubulointerstitial lesions and mortality in proteinuric mice. More importantly, the inhibition of this pathway by PBA protects kidneys from morphological and functional degradation in proteinuric mice. These results are relevant to human CKD, as LCN2 is increased in proteinuric patients. In conclusion, our study identifies a therapeutic strategy susceptible to improve the benefit of RAS inhibitors in proteinuria-induced CKD progression.

## Abstract (web summary)

Proteinuria promotes chronic kidney disease progression. Karoui *et al.* show that proteinuria stimulates overexpression of iron transporting protein lipocalin-2 via Ca^2+^ release-induced ER stress, which leads to tubular apoptosis, and that inhibition of this pathway by PBA delays renal deterioration in proteinuric mice.

## Graphical abstract

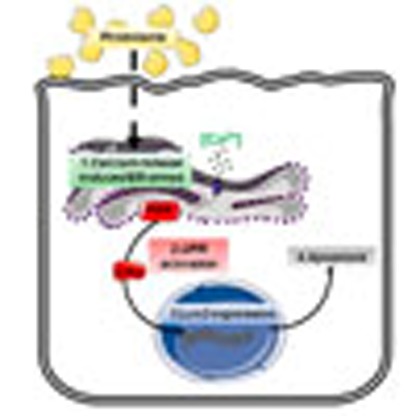

Chronic kidney disease (CKD) is characterized by a progressive decline in renal function to end-stage renal disease that can occur, irrespective of the cause of renal damage, when a critical number of functional nephrons has been lost. CKD is now a worldwide concern: >10% of the US residents suffer from this disease^1^ and similar rates are found in developing countries^2^. Understanding the pathophysiology of CKD progression is therefore a critical challenge for public health.

Most CKD are characterized by abnormalities of the glomerular filtration barrier, leading to increased glomerular permeability and abnormal filtration of macromolecules, such as albumin. Convergent evidences from clinical and experimental studies indicate that albuminuria and proteinuria are not simply markers of CKD progression, but active players in the evolution of the disease^3^. Mechanistically, it has been proposed that the proteins that escape into the glomerular filtrate have a toxic effect on tubular cells and that, once damaged, tubular cells lead to the development of interstitial fibrosis and inflammation^4^,^5^,^6^. Remarkably, several clinical studies have shown that the decline of renal function correlates more closely with the tubulointerstitial lesions than with the glomerular damage^7^. Hence, over the last 20 years, researchers have focused their efforts on the discovery of the molecular links between proteinuria and the development of tubulointerstitial lesions. Several candidates have been identified, that is, endothelin-1, MCP-1, RANTES or complement components^5^,^6^,^8^. However, so far, this did not lead to the development of novel therapeutic strategies susceptible to slow down CKD progression in humans. The only available strategy to counteract the deleterious effect of proteinuria is the renin–angiotensin system (RAS) inhibition, which reduces the leakage of proteins from the glomerular filtration barrier^9^. However, a residual proteinuria is observed in most patients under RAS blockade and the nephroprotective effect of RAS inhibitors may decline over time^10^,^11^,^12^. Moreover, attempts to further increase RAS blockade in proteinuric patients have revealed a high rate of severe side effects, such as hypotension or life-threatening hyperkalemia^13^. Thus, there is an urgent need to identify novel therapeutic targets susceptible to add benefit to RAS inhibition by preventing the toxic effect of residual proteinuria.

The endoplasmic reticulum (ER) has emerged as a signalling platform that responds to various cellular stresses by inducing a coordinated response, the unfolded protein response (UPR)^14^. During UPR, inositol-requiring enzyme 1α (IRE1α) promotes the phosphorylation of c-JUN and the specific splicing of UPR transcription factor X-box binding protein 1 (XBP1). Besides, protein kinase R-like kinase (PERK) phosphorylates eukaryotic translation-initiation factor 2α (eIF2α): this reduces general translation but promotes translation of activating transcription factor 4 (ATF4), which activates the CCAAT/enhancer-binding protein homologous protein (CHOP). If this adaptive response cannot overcome ER stress, it triggers apoptotic cell death. UPR and ER stress can be targeted by various therapeutic compounds either Food and Drug Administration approved or in preclinical studies^15^. Interestingly, a few studies showed that UPR is activated in tubular cells exposed to albumin^16^,^17^,^18^, but the pathophysiological role of such activation remains unknown^17^,^18^.

Here we combined *in vivo* and *in vitro* studies to dissect a novel molecular pathway in which albumin leads, via calcium-dependent ER stress activation, to Lipocalin 2 (LCN2) overexpression, which in turn triggers tubular cell apoptosis and renal lesions. More importantly, we showed that inhibiting ER stress with 4-phenylbutyric acid (PBA) prevents proteinuria-induced renal lesions and LCN2 overexpression.

## Results

### Proteinuria leads to UPR activation *in vitro* and *in vivo*

**Figure 1:**
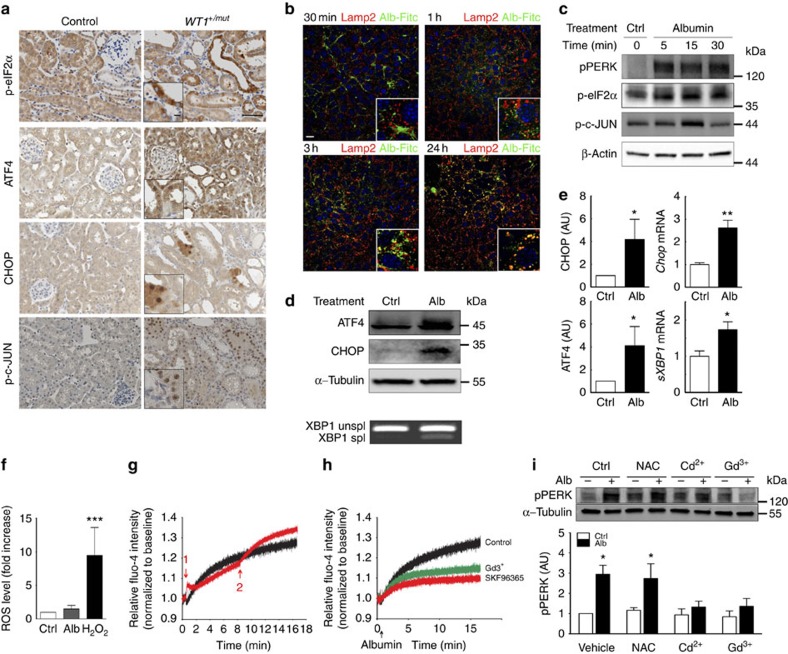
UPR is induced in tubular cells exposed to albumin *in vivo* and *in vitro.* (**a**) p-eIF2α, ATF4, CHOP and p-c-JUN evaluated in kidneys of *WT1*^*+/mut*^ mice (*n*=10) and their wild-type littermates (Control, *n*=6) 6 weeks after birth (representative panels scale bar, 100 μm; insert scale bar, 10 μm). (**b**) Internalization of extracellular albumin in mIMCD-3 cells (exposed to 1% albumin–fluorescein isothiocyanate (Alb-FITC) for 30 min and 1, 3 and 24 h) evaluated using anti-LAMP2 antibodies (red), a marker of the lysosomal compartment. Pictures show endocytosis within 30 min to 1 h of the albumin and it's targeting to the lysosomal compartment after 24 h of treatment (*n*=3; scale bar, 20 μm). (**c**) pPERK, p-eIF2α and p-c-JUN expression during time-course exposition of mIMCD-3 cells to 1% albumin (*n*=5). (**d**) ATF4 and CHOP expression in mIMCD-3 cells exposed to 1% albumin (Alb) for 24 h (upper panel) and splicing of XBP1 evaluated by reverse transcriptase–PCR (down) in mIMCD-3 cells exposed to 1% albumin for 3 h (lower panel; *n*=3). (**e**) Quantifications of ATF4 and CHOP protein abundance and mRNA of CHOP or spliced XBP1 in mIMCD-3 cells exposed to 1% albumin for 24 or 3 h, respectively (*n*=6 and *n*=5 for protein and mRNA quantifications, respectively). (**f**) Measurement by dichlorodihydrofluorescein diacetate (DCFh-DA) of intracellular ROS generation in mIMCD-3 cells exposed for 30 min to 1% albumin or 100 mM H_2_O_2_ (*n*=3). (**g**) Measurement of intracellular calcium in mIMCD-3 cells exposed to 1% albumin (black trace) or first exposed to 1.0 μM thapsigargin (red trace, red arrow 1) and then to 1% albumin (red trace, red arrow 2; *n*=3). (**h**) Measurement of intracellular calcium in mIMCD-3 cells exposed to 1% albumin for indicated times in control conditions (black trace), with gadolinium (Gd^3+^, green trace) or with SKF96365 (red trace) (*n*=3). (**i**) Representative western blotting (upper panel) and quantification (lower panel) of pPERK evaluated in mIMCD-3 cells exposed to 1% albumin for 30 min in the presence or the absence of NAC, Cd^2+^ or Gd^3+^ (*n*=5). Data are mean±s.e.m. Statistical analysis: one way analysis of variance followed by Tukey–Kramer test; Mann–Whitney test when only two groups are compared; *P<0.05, **P<0.01, ***P<0.001 versus controls.

To investigate whether ER stress is a common response to protein overload, we first studied UPR activation in several complementary models of genetic and acquired glomerular diseases. We observed that both PERK and IRE1 pathways were activated in proteinuric *WT1*^*+/mut*^ mice, which carry a mutation of *Wt1* (ref. 19), a gene encoding an essential transcription factor of podocyte homeostasis (Fig. 1a) and *Nphs2*^*Δ/−*^mice, which display dramatic podocin downregulation on tamoxifen exposure^20^ (Supplementary Fig. 1a). The activation of these pathways was confirmed in mice receiving doxorubicin (doxo) or serum bovine albumin, two widely used models of acquired proteinuric nephropathy^21^ (Supplementary Fig. 1b,c). To ascertain that UPR was the direct consequence of proteinuria, we incubated renal tubular cells with albumin. Immunofluorescence experiments using albumin–fluorescein isothiocyanate showed the progressive (from 1 h) accumulation of albumin in endocytotic vesicles of mouse inner medulla collecting duct (mIMCD-3) cells, followed by a later co-localization of albumin with lysosomal markers (LAMP2; Fig. 1b). Nevertheless, albumin exposure was associated with phosphorylation of PERK, eIF2α and c-JUN from 5 min of incubation, which was followed by the subsequent overexpression of ATF4 and CHOP, and XBP1 splicing (Fig. 1c–e).These results demonstrate that the activation of PERK and IRE1 pathways occurs before albumin internalization.

### Albumin-induced UPR activation is calcium dependent

We then investigated the mechanisms by which albumin induces UPR in tubular cells. Previous studies have demonstrated that albumin exposure leads to reactive oxygen species (ROS) production and intracellular calcium increase, two biological processes known to induce ER stress^22^,^23^. Interestingly, within 30 min of albumin exposure, whereas we failed to detect any increase of ROS (Fig. 1f), we observed a rapid increase of cytosolic calcium concentration ([Ca^2+^]_cyt_; Fig. 1g). Thapsigargin, a known inhibitor of the sarco/ER Ca^2+^-ATPase that leads to ER Ca^2+^ release, and thereafter to a Ca^2+^ influx called store-operated calcium entry (SOCE), induced a very similar [Ca^2+^]_cyt_ rise (Fig. 1g). Addition of albumin after thapsigargin stimulated a slight second [Ca^2+^]_cyt_ increase, suggesting that albumin and thapsigargin share the same pathway to mobilize Ca^2+^. To confirm the role of the SOCE in this mobilization, we repeated the same experiments in the presence of two SOCE-specific inhibitors gadolinium (Gd^3+^) and SKF96365 (ref. 24). As shown in Fig. 1h, Gd^3+^ and SKF96365 partially abolished the albumin-induced [Ca^2+^]_cyt_ rise, demonstrating that albumin induces the rise of [Ca^2+^]_cyt_ by triggering Ca^2+^ release from the ER, which in turn activates a SOCE. We then used 1,2-bis(o-aminophenoxy)ethane-*N*,*N*,*N*,*N*'-tetraacetic acid (BAPTA), a calcium-specific aminopolycarboxylic acid, to chelate the extracellular Ca^2+^. When BAPTA was added at the same time as albumin, chelation of extracellular Ca^2+^ fully prevented the [Ca^2+^]_cyt_ rise (Supplementary Fig. 2a), demonstrating that in the absence of free Ca^2+^ albumin can induce neither the Ca^2+^ release from the ER nor the subsequent SOCE. However, when we added BAPTA 5 min after albumin exposure, albumin triggered a rapid increase of [Ca^2+^]_cyt_; however, as soon as BAPTA was added, [Ca^2+^]_cyt_ started to slowly decrease (Supplementary Fig. 2a), demonstrating that albumin required extracellular Ca^2+^ to sustain the rise of [Ca^2+^]_cyt_. Depletion of Ca^2+^ from the ER compartment is known to induce ER stress and UPR activation. To demonstrate that albumin-induced Ca^2+^ release is responsible for the observed UPR activation, we treated tubular cells exposed to albumin with either *N*-acetyl-cystein (NAC), an inhibitor of ROS generation, or cadmium (Cd^2+^) and Gd^3+^, two inhibitors of Ca^2+^ transporters. Consistent with the observation that ROS did not increase after 30 min of albumin exposure, NAC treatment affected neither PERK nor eIF2α phosphorylation (Fig. 1i and Supplementary Fig. 2b). In contrast, Cd^2+^ and Gd^3+^ administration completely prevented UPR activation (Fig. 1i and Supplementary Fig. 2b), indicating that intracellular Ca^2+^ is the critical intermediate between albumin and UPR activation in this pathological setting.

### Albumin-induced UPR leads to LCN2 overexpression

**Figure 2:**
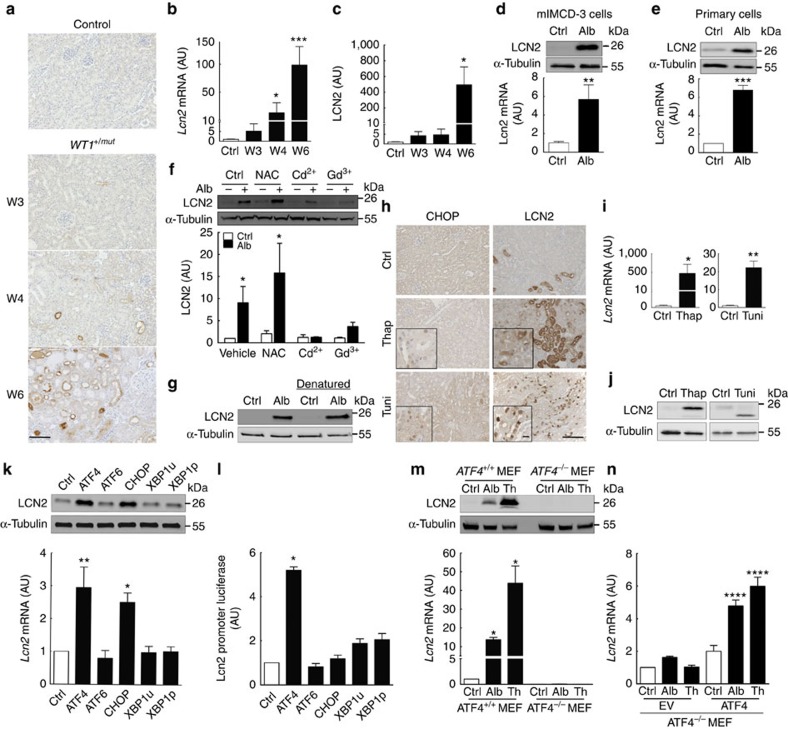
Albumin-induced UPR leads to LCN2 overexpression through ATF4. LCN2 expression evaluated by (**a**) immunohistochemistry (scale bar, 100 μm), (**b**) quantitative reverse transcriptase–PCR and (**c**) western blotting in kidneys in *WT1*^*+/mut*^ mice at 3 (*n*=5), 4 (*n*=6) and 6 (*n*=7) weeks after birth and their control littermates (*n*=5). LCN2 overexpression occurred before apparition of tubular lesions. (**d**,**e**) LCN2 protein (upper panel) and mRNA (lower panel) abundance in (**d**) mIMCD-3 cells and (**e**) in mouse renal primary cultured cells exposed to 1% albumin (Alb) for 24 h (*n*=3). (**f**) Representative western blotting (upper panel) and quantification (lower panel) of LCN2 protein abundance in mIMCD-3 cells exposed to 1% albumin for 24 h, treated with NAC, Cd^2+^ or Gd^3+^ (*n*=5). (**g**) LCN2 protein expression in mouse renal primary cultured cells exposed to 1% albumin or heat-denatured (denatured) 1% albumin for 24 h (*n*=3). (**h**) CHOP and LCN2 protein, and (**i**) *Lcn2* mRNA expression in mice injected with thapsigargin (Thap), tunicamycin (Tuni) or the vehicle (Ctrl). (*n*=5 per group; scale bar, 100 μm; insert scale bar 10 μm). (**j**) LCN2 expression in mIMCD-3 cells exposed to Thap (left panel) or Tuni (right panel) for 24 h (*n*=3). The different apparent size of LCN2 protein due to the inhibition of LCN2 glycosylation by Tuni is worth noting. (**k**) LCN2 protein (upper panel) and mRNA abundance (lower panel) in mIMCD-3 cells transiently transfected with different constructs (control, ATF4, ATF6, CHOP, unspliced XBP1 (XBP1u) and spliced XBP1 (XBP1p)). (*n*=3). (**l**) Luciferase activity in mIMCD-3 cells co-transfected with different transcription factors and a *Lcn2* promoter luciferase reporter (*n*=3). (**m**) LCN2 protein and mRNA expression in wild-type *ATF4*^*+/+*^ and *ATF4*^*−/−*^ MEFs exposed to 1% albumin or 0.5 μM thapsigargin (Th) for 24 h (*n*=3). (**n**) LCN2 mRNA expression in *ATF4*^*−/−*^ MEFs transfected with empty vector (EV) or ATF4 construct and exposed to 1% albumin or 0.5 μM thapsigargin (Th) for 24 h (*n*=3). Statistical analysis: one way analysis of variance followed by Tukey–Kramer test; Mann–Whitney test when only two groups are compared; *P<0.05, **P<0.01, ***P<0.001 versus controls.

As all these results indicated that albumin leakage from damage glomeruli leads to UPR in tubular cells, we next aimed at identifying the potential targets of UPR activation during tubulointerstitial lesion development. Among the possible candidates, we focused on LCN2 (also known as NGAL, 24p3, siderocalin or uterocalin), a small, secreted iron-transporting protein, as (i) it has been recently reported that UPR activation is associated with LCN2 overexpression in cancer cells^25^ and (ii) we previously showed that LCN2 is critically involved in the progressive deterioration of tubules following nephron reduction^26^. We observed that Lcn2 messenger RNA and protein expression progressively increased in proteinuric *WT1*^*+/mut*^ mice as compared with control littermates (Fig. 2a–c). Co-localization experiments revealed that LCN2 was expressed in tubules (mainly proximal tubules, Henle's loops and few collecting ducts) but not in glomeruli (Supplementary Fig. 3a–c). Consistent with this observation, LCN2 was not found to be increased in glomeruli from *WT1*^*+/mut*^ mice as compared with wild-type littermates (Supplementary Fig. 3a,b). These results were confirmed in the other experimental models of proteinuria (Supplementary Fig. 4a–c). More importantly, we observed that these findings were not restricted to mice: in patients with proteinuric nephropathies LCN2 immunoreactivity remarkably increased, but exclusively in tubular cells (Supplementary Fig. 5), suggesting that LCN2 overexpression is a common response to protein overload. In favour of this idea, we demonstrated that the expression of LCN2 dramatically increased when cultured tubular cells were directly exposed to albumin (Fig. 2d,e). Inhibition of calcium entry by Cd^2+^ and Gd^3+^ completely prevented LCN2 upregulation (Fig. 2f), suggesting that UPR is an essential step in albumin-induced LCN2 overexpression. On the other hand, heat denaturation experiments showed that LCN2 induction was similar, regardless of the albumin state (Fig. 2g), excluding the possibility that compounds bound to albumin account for the overexpression of LCN2, as reported for other biological outcomes^27^. Moreover, we showed that thapsigargin and tunicamycin, two compounds known to trigger ER stress, lead to a marked induction of *Lcn2* gene expression both *in vivo*, in treated mice (Fig. 2h,i), and *in vitro*, in mIMCD-3 cells (Fig. 2j), demonstrating that ER stress *per se* induces LCN2 expression.

### Albumin induces LCN2 overexpression through ATF4

We then investigated the molecular links between UPR and LCN2 expression. UPR induces several transcription factors such as ATF4, CHOP, XBP1 or ATF6 (ref. 14). To determine whether one of them is involved in LCN2 overexpression, we transfected mIMCD-3 cells with vectors encoding each of these factors and studied Lcn2 mRNA and protein expression. Interestingly, only ATF4 and CHOP were able to induce the expression of the endogenous *Lcn2* gene (Fig. 2k). However, when we used a *Lcn2*-luciferase reporter vector, we observed that ATF4, but not CHOP, significantly stimulated the *Lcn2* promoter activity (Fig. 2l), indicating that the effect of CHOP on the endogenous *Lcn2* promoter was either indirect or the result of a binding to a site located outside of the sequence used to drive the reporter gene. Consistent with the *Lcn2*-luciferase experiment, *in silico* analysis of the *Lcn2* promoter confirmed the presence of a putative ATF4 binding site located at −1,091/−1,070 upstream of the transcription start site (TSS: Chr2, 32243259). To ascertain that ATF4 is required for UPR-induced LCN2 expression, we exposed *Atf4*-null mouse embryonic fibroblast (MEF) to either albumin or thapsigargin. Experiments showed that albumin exposure induced ER stress in MEF as in tubular cells (Supplementary Fig. 6a,b). We observed that in the absence of ATF4, UPR could no more stimulate LCN2 synthesis (Fig. 2m), despite the persistent overexpression of CHOP under ER stress (Supplementary Fig. 6a). In addition, rescue experiments using an ATF4 expression vector (Supplementary Fig. 6b,c) showed that ATF4 overexpression in *Atf4*-null MEF restored the albumin- and thapsigargin-induced LCN2 expression (Fig. 2n). Taken together, these results demonstrate that ATF4 is necessary for the efficient induction of *Lcn2* gene expression during UPR.

### LCN2 deficiency protects tubular cells from proteinuria

**Figure 3:**
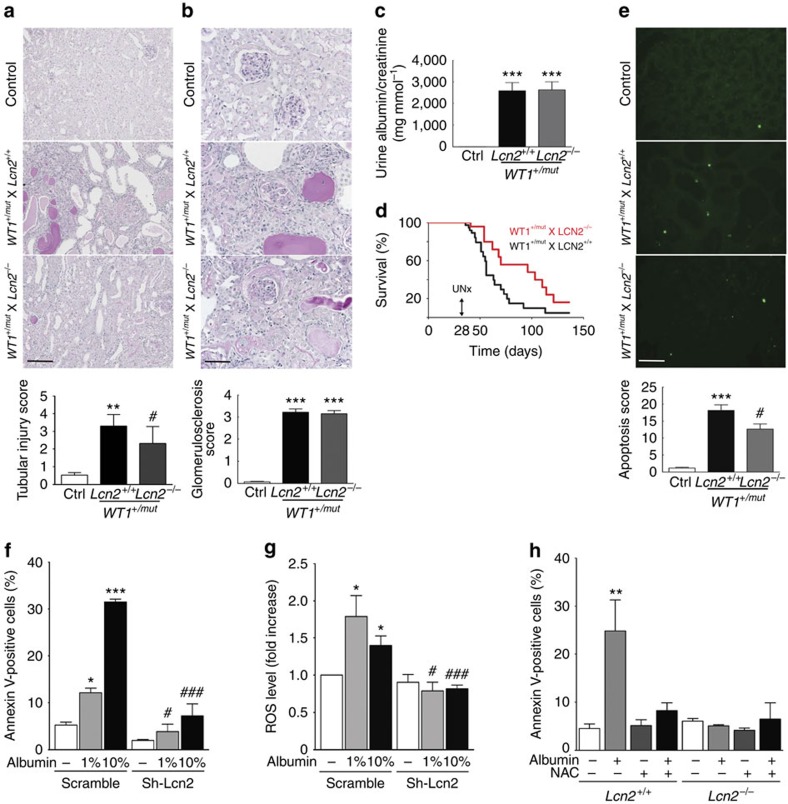
LCN2 deficiency protects from proteinuria-induced tubular lesions and apoptosis. (**a**) Renal tubular (periodic acid–Schiff (PAS); scale bar, 100 μm) and (**b**) glomerular (PAS; scale bar, 50 μm) morphology (upper panels) and quantifications (lower panels) in control (*n*=6), *WT1*^*+/mut*^*XLcn2*^*+/+*^ (*n*=10) and *WT1*^*+/mut*^*XLcn2*^*−/−*^ (*n*=10) mice, 6 weeks after birth. (**c**) Quantification of urine albumin/creatinine ratio in control (*n*=6), *WT1*^*+/mut*^*XLcn2*^*+/+*^ (*n*=5) and *WT1*^*+/mut*^*XLcn2*^*−/−*^ (*n*=10) mice at the time of killing. (**d**) Survival curve of *WT1*^*+/mut*^*XLcn2*^*+/+*^ (*n*=37) and *WT1*^*+/mut*^*XLcn2*^*−/−*^ (*n*=24) mice. (**e**) Tubular cell apoptosis evaluated using TUNEL (upper panel; scale bar, 100 μm) and quantification (lower panels) in control, *WT1*^*+/mut*^*XLcn2*^*+/+*^ and *WT1*^*+/mut*^*XLcn2*^*−/−*^ mice (*n*=6 per group). (**f**) Apoptosis measurement by annexin V staining in scramble and Lcn2 sh-RNA expressing mIMCD-3 cells exposed to albumin for 24 h (*n*=3). (**g**) ROS abundance in scramble and LCN2 shRNA expressing mIMCD-3 cells exposed to albumin for 24 h (*n*=4). (**h**) Apoptosis measurement by annexin V staining in *Lcn2*^*+/+*^ or *Lcn2*^*−/−*^ MEFs exposed to 10% albumin for 24 h in the presence of either NAC or the vehicle (PBS) (*n*=3). Data are mean±s.e.m. Statistical analysis: one way analysis of variance followed by Tukey–Kramer test; *P<0.05, **P<0.01, ***P<0.001 versus control; ^#^*P*<0.05, ^###^*P*<0.001 versus *Lcn2*^*+/+*^; log rank test (**d**) *P*<0.05.

To investigate whether LCN2 is the critical effector of this novel molecular pathway, we generated double transgenic mice in which the *Wt1* mutant allele was introduced in mice lacking the *Lcn2* gene^26^. As expected, *WT1*^*+/mut*^*XLcn2*^*+/+*^ mice developed severe renal lesions 6 weeks after birth (Fig. 3a,b). However, the frequency and the severity of tubular lesions were significantly reduced in proteinuric *WT1*^*+/mut*^ mice lacking *Lcn2* (Fig. 3a). In contrast, neither the severity of glomerular lesions (Fig. 3b) nor the level of proteinuria (Fig. 3c) was affected by *Lcn2* gene inactivation. Consistent with the persistence of severe proteinuria, the induction of UPR was similar in tubular cells of *WT1*^*+/mut*^ mice, regardless of the *Lcn2* genotype (Supplementary Fig. 7a,b). Remarkably, the improvement of kidney lesions was associated with a significant delay of the overall mortality (median survival rate 96 versus 56 days) in *WT1*^*+/mut*^*XLcn2*^*−/−*^ mice as compared with *WT1*^*+/mut*^*XLcn2*^*+/+*^ littermates (Fig. 3d). Of note, LCN2 deficiency did not prevent the development of mild interstitial fibrosis in *WT1*^*+/mut*^*XLcn2*^*−/−*^ mice at 6 weeks (Supplementary Fig. 7c).

### LCN2 deficiency reduces apoptosis via ROS modulation

We next wondered which were the cellular mechanisms involved in LCN2-induced tubular injury during proteinuria. Uncontrolled and prolonged ER stress leads to apoptosis^28^ and LCN2 has been shown to favour apoptosis in multiple cellular types^29^. As tubular cell apoptosis has been shown to play a role in CKD progression in proteinuric nephropathies^30^, we combined *in vivo* and *in vitro* experiments, to investigate whether LCN2 might regulate tubular cell apoptosis during proteinuria. *In vivo*, we observed that the number of TUNEL (TdT-mediated dUTP nick end labelling)-positive tubular cells was significantly reduced in proteinuric *WT1*^*+/mut*^ mice lacking *Lcn2* as compared with *WT1*^*+/mut*^*XLcn2*^*+/+*^ littermates (Fig. 3e). Similarly, the expression of active caspase 3, a well-known marker of apoptosis, was significantly reduced in the kidneys of *WT1*^*+/mut*^*XLcn2*^*−/−*^ mice (Supplementary Fig. 8). Consistently, *in vitro*, the number of apoptotic cells was significantly decreased in *Lcn2*-silenced tubular cells when stressed with albumin (Fig. 3f and Supplementary Fig. 9). The mechanisms of the pro-apoptotic role of LCN2 are still a matter of debate^29^. As ROS generation has been involved in ER stress-induced apoptosis^31^, we investigated whether LCN2 acts in a ROS-dependent manner in stressed cells. Our results showed that LCN2 deficiency significantly reduced ROS content in tubular cells after 24 h of albumin exposure (Fig. 3g). Consistent with this observation, *Lcn2*-silencing prevented albumin-induced overexpression of heme oxygenase 1 (*HO-1*), a gene involved in the anti-oxidative response (Supplementary Fig. 10a). Notably, when NAC was added to albumin-treated cells, apoptosis was reduced at the same extent as those in cells lacking *Lcn2* (Fig. 3h). As expected, we observed that NAC treatment prevented albumin-induced intracellular ROS accumulation (Supplementary Fig. 10b). As NAC did not inhibit LCN2 expression (Fig. 2f), we concluded that LCN2 induces apoptosis after prolonged albumin exposure by promoting ROS generation.

### PBA delays CKD progression during proteinuria

**Figure 4:**
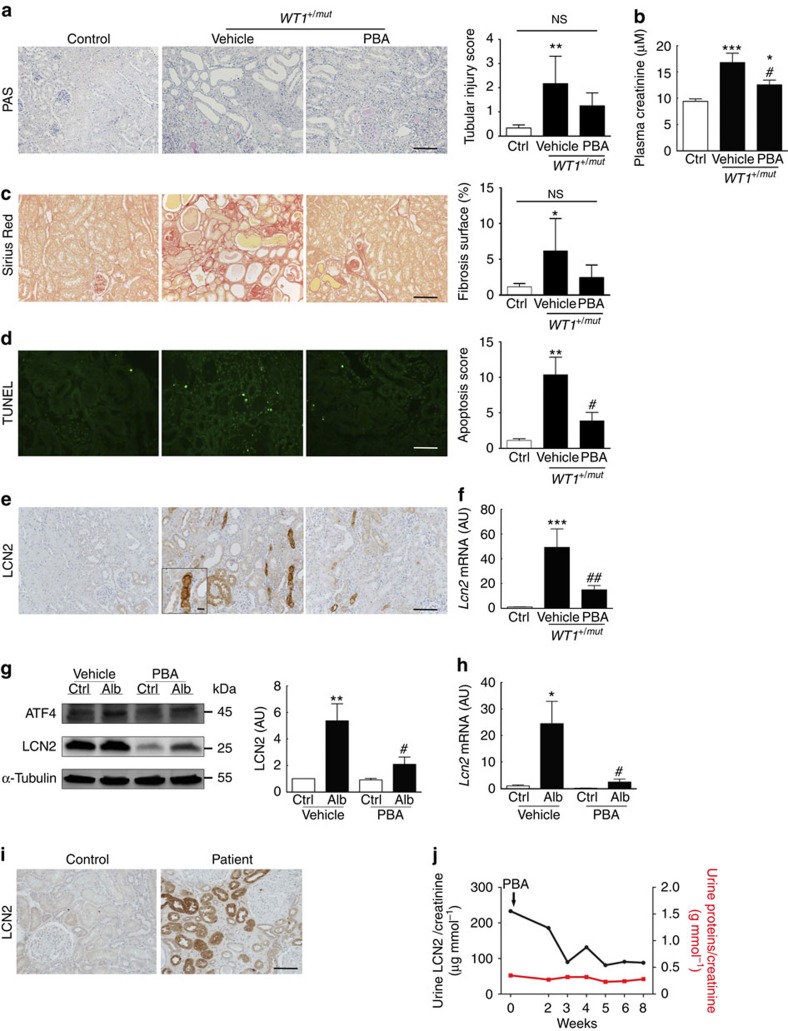
PBA protects from proteinuria-induced tubular lesion development and renal dysfunction by modulating LCN2 expression. (**a**–**e**) *WT1*^*+/mut*^ mice and wild-type littermates were treated with PBA or vehicle for 2 weeks. As no significant difference between vehicle and PBA-treated *WT1*^*+/+*^ mice was observed, only one group (Control) is represented. (**a**) Renal tubular morphology (left panels, periodic acid–Schiff (PAS) staining; scale bar, 100 μm) and quantification (right panel) in control (*n*=6), vehicle-treated *WT1*^*+/mut*^ (*n*=6) and PBA-treated *WT1*^*+/mut*^ (*n*=11) mice. (**b**) Interstitial fibrosis (left panels, Sirius red staining; scale bar, 100 μm) and quantification (right panel) in control (*n*=6), vehicle-treated *WT1*^*+/mut*^ (*n*=6) and PBA-treated *WT1*^*+/mut*^ (*n*=6) mice. (**c**) Plasma creatinine levels in control (*n*=6), vehicle-treated *WT1*^*+/mut*^ (*n*=5) and PBA-treated *WT1*^*+/mut*^ (*n*=9) mice. (**d**) Tubular cell apoptosis evaluated using TUNEL (left panel; scale bar, 100 μm) and quantification (right panel; *n*=6 per group). (**e**) LCN2 protein (scale bar, 100 μm; insert scale bar, 10 μm) and (**f**) mRNA expression in control (*n*=6), vehicle-treated *WT1*^*+/mut*^ (*n*=6) and PBA-treated *WT1*^*+/mut*^ (*n*=10) mice. (**g**,**h**) mIMCD-3 cells were exposed to 1% albumin for 24 h in the presence of either PBA or the vehicle (*n*=3). (**g**) Western blotting of ATF4 and LCN2 (left panel), and quantification (right panel; *n*=3). (**h**) *Lcn2* mRNA expression (*n*=3). (**i**) LCN2 immunohistochemistry in the renal biopsy of a proteinuric patient (before PBA administration) and a control (scale bar, 100 μm). (**j**) Evolution of urinary LCN2 excretion (black line) and proteinuria (red line) before and during PBA treatment (arrow indicates the beginning of the treatment) in the proteinuric patient. Data are mean±s.e.m. Statistical analysis: one way analysis of variance followed by Tukey–Kramer test; *P<0.05, **P<0.01, ***P<0.001 versus controls: ^#^*P*<0.05, *^##^P*<0.01 versus vehicle-treated *WT1*^*+/mut*^ mice or cells.

Several therapeutic strategies have been developed to modulate pathological ER stress, such as chemical chaperones that improve ER function, possibly by attenuating protein misfolding and stabilizing protein conformation^15^. Among them, the PBA is already used with a good safety profile in humans^32^. To provide the rational bases for a novel pharmacological strategy susceptible to counteract the toxic effect of proteinuria during CKD progression, we treated *WT1*^*+/mut*^ mice with PBA. As expected, PBA treatment resulted in a marked reduction of ER stress in kidneys of proteinuric *WT1*^*+/mut*^ mice, as judged by the decrease of p-eIF2α, ATF4, CHOP and p-c-JUN (Supplementary Fig. 11a). Remarkably, PBA administration was associated with a dramatic improvement of kidney lesions and function: tubular lesions (Fig. 4a), interstitial fibrosis (Fig. 4c) and plasma creatinine (Fig. 4b) were reduced in *WT1*^*+/mut*^ PBA-treated mice as compared with *WT1*^*+/mut*^ vehicle-treated littermates. Similarly, the number of tubular apoptotic cells was significantly reduced in *WT1*^*+/mut*^ mice receiving PBA as compared with the vehicle-treated animals (Fig. 4d). It is worth noting that as observed in *WT1*^*+/mut*^ mice lacking LCN2, the reduction of tubulointerstitial lesions was independent of the severity of glomerular injury and proteinuria (Supplementary Fig. 11b–d). Importantly, the beneficial effect of PBA was associated with a dramatic reduction of LCN2 expression in both proteinuric *WT1*^*+/mut*^ mice (Fig. 4e,f) and mIMCD-3 cells exposed to albumin (Fig. 4g,h). The decrease of LCN2 synthesis was associated with reduced ATF4 expression (Fig. 4g), suggesting that LCN2 downregulation by PBA was directly related to UPR inhibition. Consistently, PBA also prevented thapsigargin-induced LCN2 expression (Supplementary Fig. 12).

**Figure 5:**
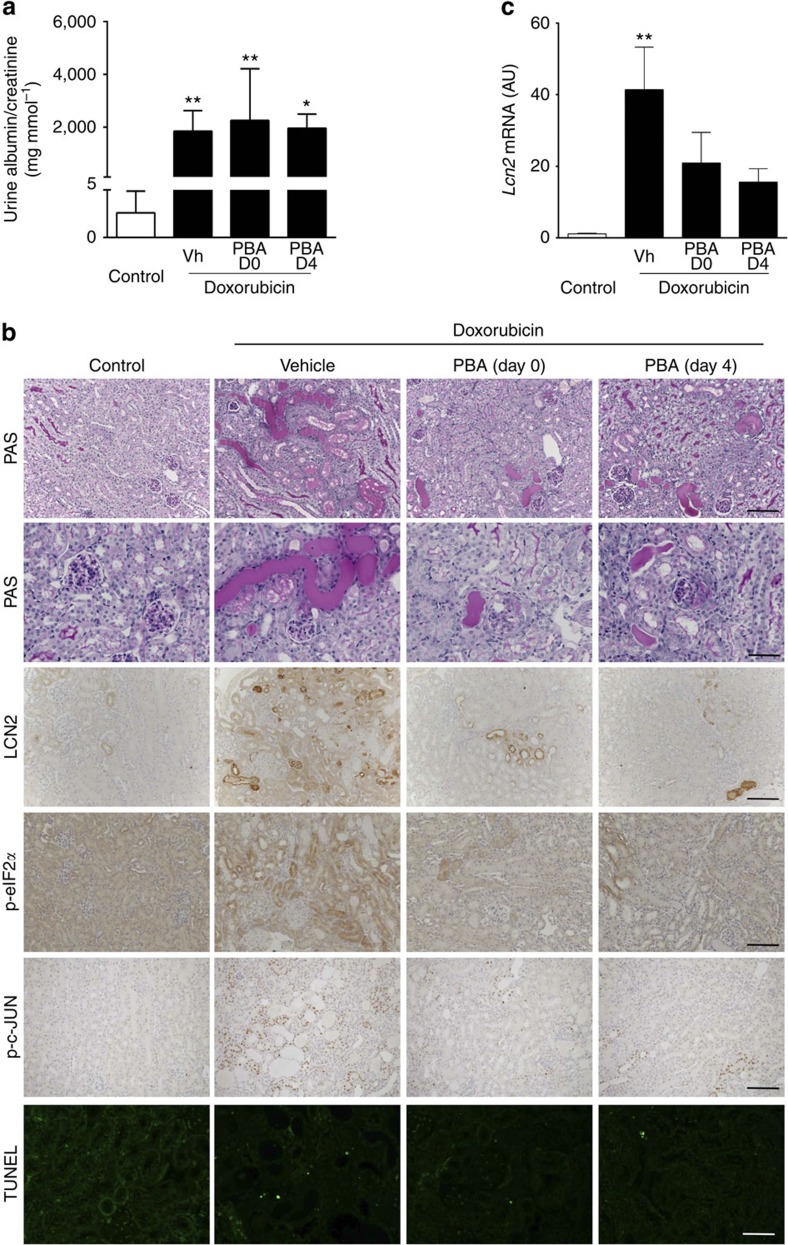
PBA decreases proteinuria-induced tubular lesions in doxo-treated mice. Doxo (12 mg kg^−1^) or saline (Control) injected mice were treated with PBA or vehicle (Vh) from day 0 (D0, preventive study) or day 4 (D4, intervention study) and killed 2 weeks after injection. As no significant difference was observed between vehicle and PBA-treated mice after saline exposure, only one group (Control) is represented. (**a**) Quantification of urine albumin/creatinine ratio at time of killing (*n*=9, *n*=10, *n*=10 and *n*=5 in control, Doxo-Vh, Doxo-PBA D0 and Doxo-PBA D4 group, respectively). (**b**) Renal tubular morphology (periodic acid–Schiff (PAS) staining; scale bar, 100 μm), glomerular morphology (PAS staining; scale bar, 50 μm), p-eIF2a, p-c-JUN and LCN2 expression (scale bar, 100 μm) and tubular cell apoptosis evaluated using TUNEL (scale bar, 100 μm; *n*=4 per group). (**c**) *Lcn2* mRNA expression (*n*=3, *n*=5, *n*=4 and *n*=5 in control, Doxo-Vh, Doxo-PBA D0 and Doxo-PBA D4 group, respectively). Data are mean±s.e.m. Statistical analysis: one way analysis of variance followed by Tukey–Kramer test; *P<0.05, **P<0.01 versus controls.

To extend these observations to a model of acquired proteinuric nephropathy, we treated doxo-injected mice with PBA from either the day of doxo injection (preventive study) or 4 days later, when proteinuria was already established (intervention study). All doxo-injected mice developed severe proteinuria 2 weeks after injection, regardless of the PBA treatment (Fig. 5a). However, the severity of tubular lesions was significantly reduced in mice receiving PBA as compared with the vehicle-treated mice, in both the preventive and the intervention study (Fig. 5b). In contrast, the severity of glomerular lesions was unchanged by PBA, regardless of the treatment schedule. Consistently, ER stress-induced LCN2 expression and tubular apoptosis were reduced in mice receiving PBA (Fig. 5b,c), confirming the potential benefit of this therapeutic approach in acquired proteinuric nephropathies.

### PBA decreases urinary LCN2 in a proteinuric patient

To finally explore the potential relationship between PBA and LCN2 in humans, we took advantage of a patient followed in our nephrology department for a lysinuric protein intolerance (a urea cycle disorder due to a *SLC7A7* gene mutation), which was associated with amyloidosis and proteinuria. PBA, which is currently used in patients with urea cycle disorders as an ammonium-scavenging agent^32^, was recommended because of persistent hyperammoniema. A renal biopsy performed before PBA treatment revealed that the amyloid deposits involved exclusively the glomeruli and not the interstitial or vascular compartments. Proteinuria was severe (2.6 g per day), despite the use of a RAS inhibitor. Immunohistochemistry revealed that LCN2 expression was remarkably increased in renal tubular cells (Fig. 4i). Urinary LCN2 excretion was also dramatically increased as compared with healthy controls. Remarkably, serial urine collections revealed that PBA treatment resulted in a progressive reduction of urinary LCN2 excretion, despite sustained levels of residual proteinuria (Fig. 4j).

## Discussion

**Figure 6:**
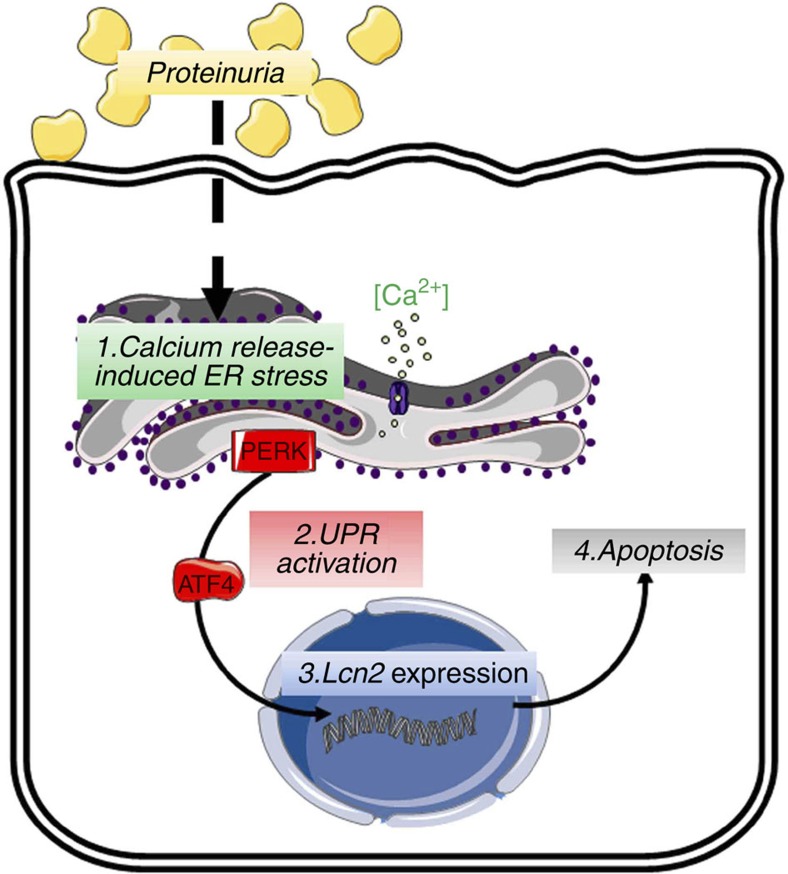
Proposed model of proteinuria-induced tubulointerstitial damage. This study shows that by inducing Ca^2+^-dependent ER stress, albumin activates ATF4 expression, which in turn stimulates LCN2 production. LCN2 then leads to increased apoptosis and tubulointerstitial damage.

Proteinuria is now recognized not simply as a marker but also as a major risk factor of CKD progression. Despite the fact that RAS inhibitors reduce the leakage of proteins in some patients, therapeutic strategies capable of counteracting the deleterious effect of residual proteinuria on the tubulointerstitial compartment are still lacking. By combining experimental models of proteinuric nephropathies with *in vitro* and *in vivo* genetic and pharmacological approaches, we uncovered a novel ER stress pathway. We demonstrated that proteinuria stimulates, via a calcium release-induced ER stress, the overexpression of LCN2, which in turn leads to tubular apoptosis and renal lesions (Fig. 6). More importantly, we showed that inhibition of this pathway by PBA, a pharmacological strategy already used in humans, delayed renal deterioration in proteinuric mice. Collectively, these data identify a novel therapeutic strategy and suggest a crucial role for albumin/ER stress/LCN2 pathway in modulating the progression of CKD.

Our study showed that ER calcium release is a crucial event in the signalling pathway leading to albumin-induced ER stress. Indeed, we observed that extracellular albumin rapidly induces an ER calcium release followed by a store-operated calcium entry that maintains the cytosolic calcium increase. Interestingly, a previous study showed that albumin induces ER stress and [Ca^2+^]_cyt_ increase in cultured podocytes through TRPC6 (transient receptor potential cation channel, subfamily C, member 6) activation^33^. Whether TRPC6, which is expressed in tubular cells^34^, plays a role in our experimental model remains to be elucidated. Furthermore, our experiments using BAPTA revealed that albumin requires extracellular calcium to induce ER calcium release. Taking all these data together, it is tempting to speculate that albumin binds to a protein receptor in a calcium-dependent manner, to trigger the intracellular events responsible for ER calcium release. Interestingly, LRP2/Megalin, a well-known albumin receptor, binds its ligands in an extracellular calcium-dependent manner^35^,^36^. It is worth noting that LRP2 is expressed in mIMCD3 cells (Supplementary Fig. 13a,b).

In the present study, we showed that AFT4 is mandatory for the efficient induction of *Lcn2* gene expression in albumin-overloaded tabular cells. In fact, albumin failed to induce *Lcn2* in *Atf4*^*−/−*^ cells and overexpression of ATF4 completely rescued the phenotype. These data are consistent with the observation that ATF4 directly binds to the *Lcn2* promoter^31^. A previous study has reported that CHOP is able to bind to the *Lcn2* promoter as well and is able to induce LCN2 expression, at least, in human cancer cells^37^. As a recent work has shown that CHOP and ATF4 interact to induce target genes during UPR^31^, it is possible that ATF4 and CHOP cooperate to induce LCN2 expression in albumin-overloaded tubular cells.

We previously showed that LCN2 plays a crucial role in CKD progression during nephron reduction and cystogenesis, by mediating the mitogenic effect of epidermal growth factor receptor^26^. In the present study, we demonstrated that LCN2 acts rather by triggering apoptosis in protein-overloaded tubular cells. Interestingly, albumin overload did not lead to epidermal growth factor receptor phosphorylation (Supplementary Fig. 14), suggesting that LCN2 might act as a central integrator of multiple signalling pathways leading to CKD progression. We also observed that LCN2 triggers apoptosis through ROS generation. The mechanisms by which LCN2 promotes oxidative stress in this pathological setting are still unknown. It is possible that an increase in intracellular iron content plays a role, as LCN2 has been shown to transport iron and modulate mitochondrial apoptosis via Bcl-2-like protein 11 (BIM) expression^38^,^39^. In favour of this idea, LCN2 endocytosis has been shown to increase ROS generation through an intracellular accumulation of iron leading to apoptosis^40^. Alternatively, LCN2 might act by inhibiting the nuclear factor-erythroid 2 p45-related factor 2 antioxidant pathway, as LCN2 has been also shown to sequestrate catechol^41^, a small metabolite product that could activate nuclear factor-erythroid 2 p45-related factor 2 (ref. 42). Whether the secreted or the intracellular form of LCN2 is involved in ROS-induced tubular apoptosis remains to be elucidated.

The substantial increase of the survival rate in proteinuric *WT1*^*+/mut*^ mice lacking LCN2 might appear bigger than expected, given the difference in renal function. However, the experimental design of the study could explain, at least in part, this discrepancy. In fact, kidney lesions and apoptosis were evaluated at 6 weeks, whereas the difference in mortality rate was discernable 4 weeks later. Considering the natural history of CKD progression, it is very likely to be that both kidney lesions and renal failure were much greater at this time point than at 6 weeks. In favour of this idea, it has been shown that this model progresses very rapidly towards the complete destruction of the kidney^19^. Nevertheless, as we studied mice bearing a germline *Lcn2* inactivation, we cannot rule out the possibility that LCN2 deficiency affected the lifespan of *WT1*^*+/mut*^ mice via a systemic effect. It is known that CKD is associated with increased cardiovascular mortality^43^. On the other hand, LCN2 has been shown to modulate vascular and cardiomyocyte functions^44^. Whether LCN2 inhibition might reduce the cardiovascular lesions and mortality associated with CKD progression is an interesting question that deserves further investigation.

Urinary LCN2 excretion has been shown to be a valuable biomarker of CKD progression^45^, including in proteinuric nephropathies^46^. Despite the fact that proteinuria is now recognized as a major risk factor of CKD progression, the rate of progression varies among proteinuric patients^47^. By showing that LCN2 is a critical target of proteinuria, our data suggest that urinary LCN2 could be helpful to mark the proteinuric patients with the higher risk of CKD progression.

Chemical chaperones have recently received increasing interest as a novel therapeutic approach for the treatment not only of rare genetic diseases leading to misfolded proteins but also of more frequent diseases, that is, metabolic diseases or cancers, characterized by ER stress^48^. Besides, multiple new therapeutic compounds targeting specific UPR branches are currently under development^15^. In the present study, we showed that pharmacological modulation of ER stress is an efficient treatment for preserving renal function and morphology in proteinuric mice, at least in part, by decreasing LCN2 expression. The observation, however, that PBA administration, but not LCN2 deficiency, decreased the severity of interstitial fibrosis in *WT1*^*+/mut*^ mutant mice suggests that PBA might act by modulating additional actors of CKD progression. Notably, the benefit was observed despite the persistence of severe proteinuria. More importantly, our clinical observation that PBA dramatically reduced LCN2 overexpression in a proteinuric patient suggests that this treatment might be valuable in humans. Although we are aware that these promising results must be confirmed, they provide new therapeutic perspectives for patients who display residual proteinuria despite RAS inhibition^11^ or develop adverse side effects on RAS inhibitors^13^.

In conclusion, by identifying a novel therapeutic strategy able to counteract the toxic effect of proteinuria on tubular cells, our study provides the rational for the development of an innovative ready-to-use multitarget therapy for proteinuric CKD patients, susceptible to inhibit not only proteinuria leakage but also its tubular toxicity. We expect that adding molecular chaperones will significantly improve the benefit of RAS inhibitors in delaying the progression of CKD, a major public health problem.

## Methods

### Animals

Mice were on FVB/N background unless otherwise specified. Mutant mice used for these studies were as follows: (i) mutant *Wt1* knock-in mice (*WT1*^*+/mut*^ mice) bearing a heterozygote *Wt1* mutation^19^, (ii) mutant *Nphs2* mice (*Nphs2*^*Δ/−*^mice), bearing a floxed *Nphs2* exon 2 allele, a-null *Nphs2* allele and a podocyte-expressed, tamoxifen-responsive Cre recombinase^20^, and (iii) *Lcn2*^*−/−*^ mice^26^. Animals were fed *ad libitum* and housed at constant ambient temperature in a 12-h light cycle. Animal procedures were approved by the Departmental Director of ‘Services Vétérinaires de la Préfecture de Police de Paris' and by the ethical committee of the Paris Descartes University (approval number: A75-15-34).

For the *WT1*^*+/mut*^ mice experiments, male and female mutant mice (*n*=10) and their control littermates (*n*=6) were uninephrectomized at 4 weeks after birth and killed 2 weeks later. For the time-course experiments, mutant *WT1*^*+/mut*^ mice were killed at 3 (*n*=5), 4 (*n*=6) and 6 (*n*=7) weeks after birth. Data were compared with a group of control littermates (*WT1*^*+/+*^ mice, *n*=5). PBA (1 g kg^−1^ per day of absorbed PBA, Merck) or vehicle (water corrected for pH and Na concentration with HCl and NaCl) was given in drinking water after uninephrectomy (UNx) performed at 4 weeks and mice were killed 2 weeks later (*n*=6, *n*=6, *n*=6 and *n*=11 in vehicle-treated *WT1*^*+/+*^, PBA-treated *WT1*^*+/+*^, vehicle-treated *WT1*^*+/mut*^ and PBA-treated *WT1*^*+/mut*^ group, respectively).

For *WT1*^*+/mut*^*XLcn2*^*−/−*^ mice experiments, male and female double mutants and their control littermates (*WT1*^*+/+*^) were uninephrectomized at 4 weeks after birth and killed 2 weeks later (*n*=6, *n*=10 and *n*=10 for *WT1*^*+/+*^, *WT1*^*+/mut*^*XLcn2*^*+/+*^ and *WT1*^*+/mut*^*XLcn2*^*−/−*^ groups, respectively). For survival experiments, male and female double mutant mice were followed up to 23 weeks after birth (*n*=37 and *n*=24 in *WT1*^*+/mut*^*XLcn2*^*+/+*^ and *WT1*^*+/mut*^*XLcn2*^*−/−*^ mice, respectively).

For *Nphs2* mice, experiments were performed on 6-week-old male and female triallellic *Nphs2*^*lox/−*^*,Cre*^*+*^ mice and their control littermates *(Nphs2*^*lox/−*^*,Cre*^*_*^ mice). Cre recombinase was induced by intraperitoneal administration of tamoxifen (33 mg kg^−1^ per day for 5 days; Sigma) and mice were killed 3–4 weeks after the last injection (*n*=6 for each genotype).

For the doxo experiments, Balb/c females were injected intravenously with doxo (10 or 12 mg kg^−1^; Sigma) or vehicle (saline) and killed either 14 or 25 days after injection (*n*≥5 per group). PBA or vehicle was given in drinking water as described above, either from the day of doxo injection (Day 0, preventive study) or from 4 days after doxo injection (Day 4, intervention study), and mice were killed 2 weeks later (*n*=6, *n*=10, *n*=10 and *n*=5 in control, vehicle-treated doxo-injected, PBA-D0 doxo-injected and PBA-D4 doxo-injected group, respectively).

For BSA experiments, FVB/N females were uninephrectomized at 12 weeks and then intraperitoneally injected with BSA (25 mg g^−1^; Sigma) or vehicle (saline) for 4 weeks (*n*=4 per group).

For the thapsigargin and tunicamycin experiments, mice were injected with thapsigargin (0.5 μg g^−1^; Sigma), tunicamycin (1 μg g^−1^; Sigma) or vehicle (dimethyl sulfoxide) and killed 24 or 48 h later for thapsigargin and tunicamycin, respectively (*n*=5 per group).

For all the studies, urine and plasma samples were collected at the time of killing and the kidneys were removed for morphological, protein and mRNA studies.

### Cell cultures

mIMCD-3 cells were cultured as previously described^26^. Cells were maintained in DMEM medium /HamF12 (1:1; Gibco, France) medium containing 10% fetal bovine serum (Sigma). *Aft4*^−/−^ mouse embryonic fibroblasts (MEFs; a kind gift of Dr David Ron) and *Lcn2*^−/−^ MEF (a kind gift from Dr Xiaoli Chen) were maintained in DMEM (Gibco) medium containing non-essential amino-acid and 10% fetal bovine serum (Sigma). Subconfluent cells were starved for 16 h and then stimulated for 24 h with 1% filtered fatty acid-free BSA (Roche) diluted in PBS. Tunicamycin (0.5 μg ml^−1^; Sigma) and thapsigargin (0.5 μM; Sigma) were used for 16–24 h. For heat-denatured albumin experiments, fatty acid-free BSA was diluted in DMEM and heated for 5 min at 95 °C. For treatment (Gd^3+^, Cd^2+^, SKF96365, NAC and PBA) experiments, mIMCD-3 cells were first starved for 6 h. For Gd^3+^ (1 μM; Sigma), Cd^2+^ (1 μM; Sigma), SKF96365 (1 μM; Sigma) and BAPTA (10 mM; Sigma), the treatment was added at the same time than 1% BSA. BAPTA was added 5 min after 1% BSA incubation, in another set of experiments. For NAC (10 mM; Sigma) and PBA (2.5 mM; Merck), the treatment was added 30 min before 1% BSA incubation. Cells were lysed in RIPA buffer for protein extraction, whereas mRNA was extracted with the Macherey–Nagel mini-kit, according to the manufacturer's protocol. All cell lines were mycoplasma free.

Primary cultures of renal cortical tubular cells were prepared as previously described^49^. Briefly, kidney cortex tubules from 3- to 4-week-old mice were micro-dissected, dissociated by collagenase treatment, washed in Hank's solution by centrifugation and filtered to obtain an enriched preparation of dissociated tubular cells. Cells were then cultivated to reach subconfluency. Cell cultures were exposed to 1% fatty acid-free BSA for 24 h and then cells were lysed in RIPA buffer for protein extraction.

Transient transfections were performed using Lipofectamine 2000 (Invitrogen) according to the manufacturer's instructions. The following plasmids were used: ATF4 (Addgene Inc., ID 21845), ATF6 (Addgene Inc., ID 11975), CHOP (Addgene Inc., ID 21898), XBP1u (Addgene Inc., ID 21832) and XBP1p (Addgene Inc., ID 21833). pcDNA3.1 vector was used as a control. After 36 h of transfection, cells were harvested for mRNA or protein preparation.

For stable cell lines, mIMCD-3 cells expressing short hairpin RNA (shRNA) against Lcn2 were obtained as follows. Double-stranded oligos encoding shRNA directed against *Mus musculus* Lcn2 were cloned into a lentiviral Tet-pLKO-puro plasmid (pLKO-Tet-on, Addgene 21915). Two double-stranded oligos were inserted between the Age1/EcoR1 restriction site of pLKO-Tet-on, to generate inducible RNA interference (RNAi) expression following doxycycline exposure (100 ng ml^−1^): scramble (TRCN0000072181) RNAi: sense: 5′-CCGGACAACAGCCACAACGTCTATACTCGAGTAT AGACGTTGTGGCTGT TGTTTTTTG-3′, antisense: 5′-AATTCAAAAAACAACAG CCACAACGTCTATACTCGAGTATAGACGTT GTGGCTGTTGT-3′; and Lcn2 (TRCN0000055328): sense: 5′-CCGGTGCCACTCCATCTTTCCTGTTCTCGAGAACAGGAAAGATGGAGTGGCATTTTTG-3′, antisense: 5′-AATTCAAAAATGCCACTCCATCTTTCCTGTTCTCGAGAACAGGAAA GATGGAGTGGCA-3′. The lentiviral particles were produced by co-transfection of HEK293T cells with three plasmids (pMD2G, psPAX2 and the shRNA vector) using Lipofectamine 2000 (Life Technologies). Cells were infected in the presence of 8 μg ml^−1^ polybrene overnight and were selected 2 days after viral transduction in puromycin (2 μg ml^−1^), to achieve 100% positive cells. For mIMCD-3 stable cell experiments (expressing scramble or sh-Lcn2 RNAi), cells were exposed to medium containing 100 ng ml^−1^ doxycycline before treatment.

### Transfections and Luciferase reporter gene assays

Luciferase gene reporter assays were carried out in 24-well plates. In brief, subconfluent mIMCD-3 cells were co-transfected with pGL3 control vector or pGL3–24p3/Lcn2-Luc containing murine *Lcn2* gene promoter (Addgene Inc., ID 25463) in combination with plasmids expressing protein of interest. pRL-SV40 *Renilla* control vector (Promega) was co-transfected in each condition. The luciferase activity was measured 36 h after transfection using a dual-luciferase reporter assay system (Promega) as recommended. Luciferase activity in each cellular lysates was normalized to *Renilla* luciferase activity. Results are presented as changes of transactivation of the murine LCN2 promoter constructs relative to the original activity of this promoter constructs in cells transfected with empty vector control plasmid.

### Clinical samples

Renal biopsies were routinely performed in proteinuric (>1 g per day) patients with diabetic nephropathy, membranous nephropathy or IgA nephropathy (*n*=3 per group). Renal biopsies from patients with resolute kidney injury that did display neither significant proteinuria nor histological lesions were used as controls.

The patient treated with PBA was a 33-year-old man, who developed proteinuric CKD associated with lysinuric protein intolerance. Blood pressure was 116/84 mm Hg, measured glomerular filtration rate (iohexol clearance) was 51 ml min^−1^ per 1.73 m^2^, serum albumin levels were 38 g l^−1^ and proteinuria 2.6 g per day. The patient was treated with irbesartan, hydrochlorothiazide, citrullin and sodium benzoate, and successively PBA (3 g per day) was added. Morning spot urine samples were obtained before PBA and every week after the beginning of the treatment for 2 months. Urine samples were stocked at −80 °C after centrifugation. No other therapeutic modification was made once PBA was introduced. Informed consent was obtained from all patients. Our institutional review board approved the study (approval number: DC-2009-955).

### Urine and plasma analysis

For mice samples, urinary albumin and creatinine levels were measured using an Olympus multiparametric autoanalyser (Instrumentation Laboratory). Plasma creatinine was measured using Konelab Analyzer (Thermo Fischer Scientific).

For human samples, urinary LCN2 concentration was evaluated by enzyme-linked immunosorbent assay (R&D Systems) according to the manufacturer's instructions. Proteinuria and urinary creatinine was measured using a Hitachi 917 analyser (Roche Diagnostics).

### Renal morphology

For morphology analysis, kidneys were fixed in 4% paraformaldehyde, paraffin embedded and 4 μm sections were stained with periodic acid–Schiff or Sirius red. The degree of glomerular lesion was evaluated using a semiquantitative score methodology as previously described^50^. The degree of tubular lesions was evaluated using semiquantitative score methodology as previously described^51^, with minor modifications in the evaluation of kidney lesions (0=normal, 1=involvement of <10% of the cortex, 2=involvement of 10–25% of the cortex, 3=involvement of 25–50% of the cortex, 4=involvement of >50% of the cortex). The degree of fibrosis was automatically quantified using Sirius red staining in eight representative fields (magnification × 100) with a Nikon digital camera Dx/m/1200 and NIS software (Nikon), and expressed as the ratio between the Sirius red-positive surface and the total section area, as previously described^26^.

### Immunohistochemistry

For mouse samples, 4-μm sections of paraffin-embedded kidneys were incubated with the following: a goat anti-mouse LCN2 antibody (R&D Systems, AF1857) at 1:100, a rabbit anti-mouse CHOP (Santa Cruz Biotechnology, SC-575) at 1:50, a rabbit anti-mouse ATF4 at 1:50 (Santa Cruz Biotechnology, SC-200), a rabbit anti-mouse p-c-JUN (Cell Signaling Technology, 2361) at 1:200, a rabbit anti-mouse p-eIF2α (Cell Signaling Technology, 3597) at 1:50, followed by a rabbit anti-goat biotinylated antibody (Dako, E0466) at 1:200, a donkey horseradish peroxidase (HRP)-conjugated anti-rabbit (GE Healthcare, NA934V) at 1:200 or a donkey biotinylated anti-rabbit antibody (GE Healthcare, RPN1004V) at 1:200. Biotinylated antibodies were detected using HRP-labelled streptavidin (Southern Biotech, 7100-05) at 1:2,000 and 3-3'-diamino-benzidine-tetrahydrochloride (Dako, K3468).

For human samples, 4 μm sections of paraffin-embedded kidneys were incubated with a goat anti-human LCN2 antibody (R&D Systems, AF1757) at 1:100, followed by a HRP-labelled rabbit anti-goat antibody (Dako, P0449) and 3-3'-diamino-benzidine-tetrahydrochloride revelation.

For co-localization experiments, lotus tetragonolobus lectin was detected using biotinylated-lotus tetragonolobus lectin (Vector, B1325) at 1:50, followed by HRP-labelled streptavidin at 1:2,000. For Tamm–Horsfall staining, a goat anti-Tamm–Horsfall antibody (AbDSerotec, 8595-0054) at 1:200 was used, followed by a biotinylated goat antibody (Dako) at 1:500 and HRP-labelled streptavidin at 1:2,000. For aquaporin (AQP2) 2 staining, sections were incubated with a rabbit anti-AQP2 antibody (Sigma, A7310) 1:400, followed by a donkey HRP-conjugated anti-rabbit antibody (GE Healthcare, NA934V) at 1:300.

Apoptosis was detected in 4 μm sections of paraffin-embedded kidneys by TUNEL assay using the In Situ Cell Death Detection kit (Roche) according to the manufacturer's protocol. The number of apoptotic cells was determined as the number of TUNEL-positive nuclei per tubule in eight representative fields (magnification × 400).

### Albumin uptake in mIMCD-3 cells

Cells exposed to 1% fluorescein isothiocyanate–albumin (Sigma) for different time were exposed to rat anti-LAMP2 antibody (Abcam, Ab 13524). The secondary antibody was fluorescent goat anti-rat antibody (Invitrogen). Images were acquired using a Leica TCS SP5 AOBS microscope with a 40/1.25 (differential interference contrast (DIC)) (Leica Microsystems). Resulting digital images were acquire with LAS AF software and analysed using ImageJ.

### Cell death measurement

Cellular death was assessed based on propidium iodide (PI) staining (Sigma) and Annexin V/PI staining (Molecular Probes) according to the manufacturer's protocols. Cell fluorescence was measured by flow cytometry. PI-negative cells were defined as viable. Flow cytometry analysis was performed on a FACSCalibur (BD Biosciences) followed by analysis using FlowJo.

### mRNA analysis

mRNAs were quantified in mouse kidneys and cultured cells by quantitative reverse transcriptase–PCR using an ABI PRISM 7700 Sequence Detection system (Applied Biosystems). Primers were as follows: Lcn2 (fwd) 5′-GGACCAGGGCTGTCGCTACT-3′ and (rev) 5′-GGTGGCCACTTGCACATTGT-3′; CHOP (fwd) 5′-GGAGGTCCTGTCCTCAGATGAA-3′ and (rev) 5′-GGACGCAGGGTCAAGAGTAGTG-3′; spliced XBP1 (fwd) 5′-GAGTCCGCAGCAGGTG-3′ and (rev) 5′-GTGTCAGAGTCCATGGGA-3′; and HO-1: (fwd) 5′-GTACACATCCAAGCCGAGAA-3′ and (rev) 5′-TGGTACAAGGAAGCCATCAC-3′.

The splicing of XBP-1 mRNA was analysed by PCR using the following protocol: 94 °C for 3 min, 29 cycles of 94 °C for 30 s, 58 °C for 30 s, 72 °C for 30 s and 72 °C for 3 min. We used the following primers: XBP-1 (fwd) 5′-ACACGCTTGGGAATGGACAC-3′ and (rev) 5′-CCATGGGAAGATGTTCTGGG-3′.

RPL13, SDHA and HPRT were used as the normalization controls as previously described^52^.

### Western blotting

Western blottings were performed as previously described^26^ using the following: a goat anti-mouse LCN2 antibody (R&D systems) at 1:1,000, a rabbit anti-mouse CHOP antibody (Santa Cruz Biotechnology) at 1:500, a rabbit anti-mouse p-c-JUN antibody (Cell Signaling Technology) at 1:1,000, a rabbit anti-mouse pPERK antibody (Cell Signaling Technology), a rabbit anti-mouse ATF4 antibody (Santa Cruz Biotechnology) at 1:500, a rabbit anti-mouse p-eIF2α antibody (Cell Signaling Technology) at 1:1,000 and a rabbit anti-mouse cleaved caspase 3 antibody (Cell Signaling Technology) at 1:1,000, followed by either a rabbit HRP-conjugated anti-goat antibody at 1:10,000 (Dako) or a donkey HRP-conjugated anti-rabbit antibody at 1:10,000 (Amersham). Mouse monoclonal anti-α-tubulin or anti-β-actin antibody (Sigma) was used as loading control. The uncropped versions of western blottings are shown in Supplementary Fig. 15.

### ROS measurement

Two techniques were used to measure intracellular ROS content. mIMCD-3 cells were loaded with dichlorodihydrofluorescein diacetate (10 μM; LifeTechnologies) and incubated for 30 min at 37 °C. After incubation, cells were washed twice with PBS, lysed and acquired on a microplate reader (Tristar LB 941, Berthold). For analysis of intracellular ROS content, CellROX Deep Red Reagent was used according to the manufacturer's instructions (LifeTechnologies) and analysed either by immunofluorescence or flow cytometry. Flow cytometry analysis was performed on a FACSCalibur (BD Biosciences) followed by analysis using FlowJo.

### Calcium spectrofluorimetry

Intracellular Ca^2+^ concentration variations were studied using spectrofluorimetry. Recordings were made in a HEPES buffered saline medium (HBS) containing (in mM): 135 NaCl, 5.9 KCl, 2 CaCl_2_, 1.2 MgCl_2_, 11.6 Hepes, 11.5 glucose pH 7.3 adjusted with NaOH. mIMCD-3 cells were grown on glass coverslips in Petri dishes. Before confluence, coverslips were washed three times with HBS medium and then loaded with 4 μM Fluo-4 AM (Molecular Probes). After loading, coverslips were washed twice with Fluo-4 AM-free HBS medium and maintained in this medium. Just before recordings, a coverslip was introduced in a 1 cm width—3 ml quartz cuvette, containing 2 ml of HBS medium and inserted in a spectrofluorimeter (RF-1501 Shimadzu Corporation, Japan). Fluo-4 was excited at 494 nm and fluorescence was measured at 516 nm. The baseline fluorescence was recorded during 10 min before adding BSA. The [Ca^2+^]_cyt_ was expressed as a ratio value of the fluorescence intensity divided by the baseline average fluorescence intensity.

### Data analysis and statistics

Data were expressed as means±s.e.m. Differences between the experimental groups were evaluated using analysis of variance followed by the Tukey–Kramer test. The log-rank test was used for survival analysis. When only two groups were compared, Mann–Whitney test was used. Assumptions for statistical analyses were met (that is, normal distribution and equal variance). Replicates used were biological replicates, which were measured by using different samples derived from distinct mice. The results are representative examples of more than three independent experiments. We estimated the sample size considering the variation and mean of the samples. No randomization or blinding was done. No animals were excluded from the analysis. The statistical analysis was performed using Graph Prism Software.

## Additional information

**How to cite this article:** El Karoui, K. *et al.* Endoplasmic reticulum stress drives proteinuria-induced kidney lesions via Lipocalin 2. *Nat. Commun.* 7:10330 doi: 10.1038/ncomms10330 (2016).

## Supplementary Material

Supplementary Information Supplementary Figures 1-15
