# Supplementary material for: Endoplasmic reticulum stress drives proteinuria-induced kidney lesions via Lipocalin 2
Source: Nat Commun. 2016 Jan 20;7:10330. doi: 10.1038/ncomms10330 (PMC4735759; doi:10.1038/ncomms10330)
Supplement: Supplementary Information — Supplementary Figures 1-15 [file ncomms10330-s1.pdf]

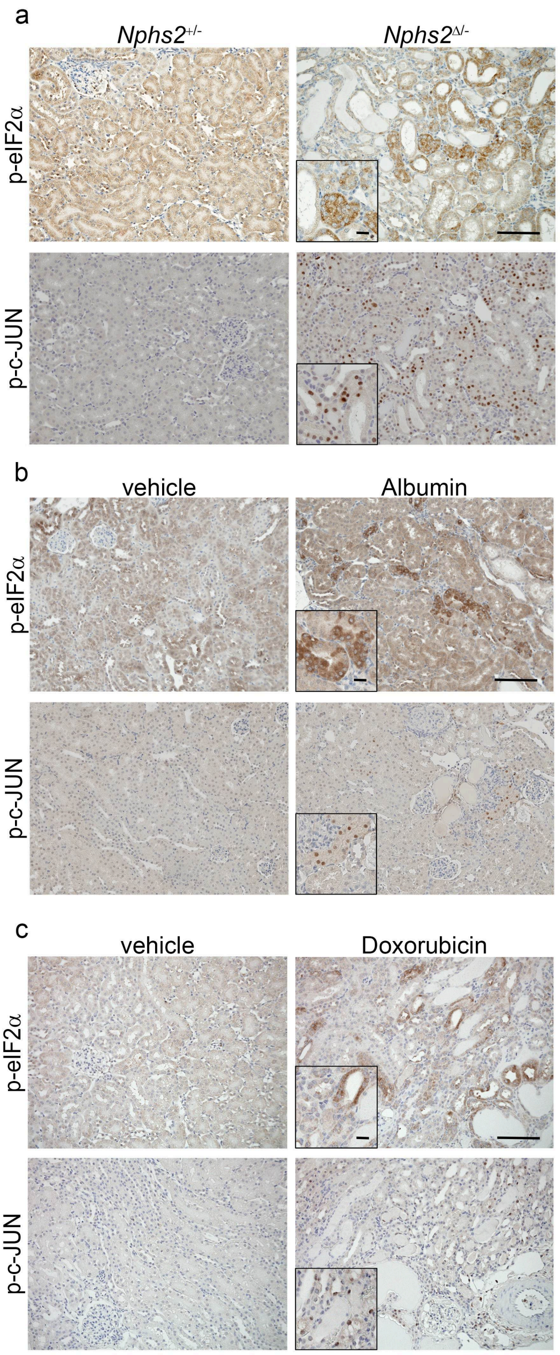

Supplementary Figure 1: ER stress is induced in tubular cells in different experimental models of proteinuria. p-eIF2a and p-c-JUN evaluated in kidneys of **(a)** *Nphs2*$^{\Delta/-}$ mice and their control littermates, **(b)** albumin and vehicle-injected mice, and **(c)** doxorubicin and vehicle-injected mice. Panels are representative samples of n = 4 per group, scale bar: 100 μm, insert scale bar: 10 μm.

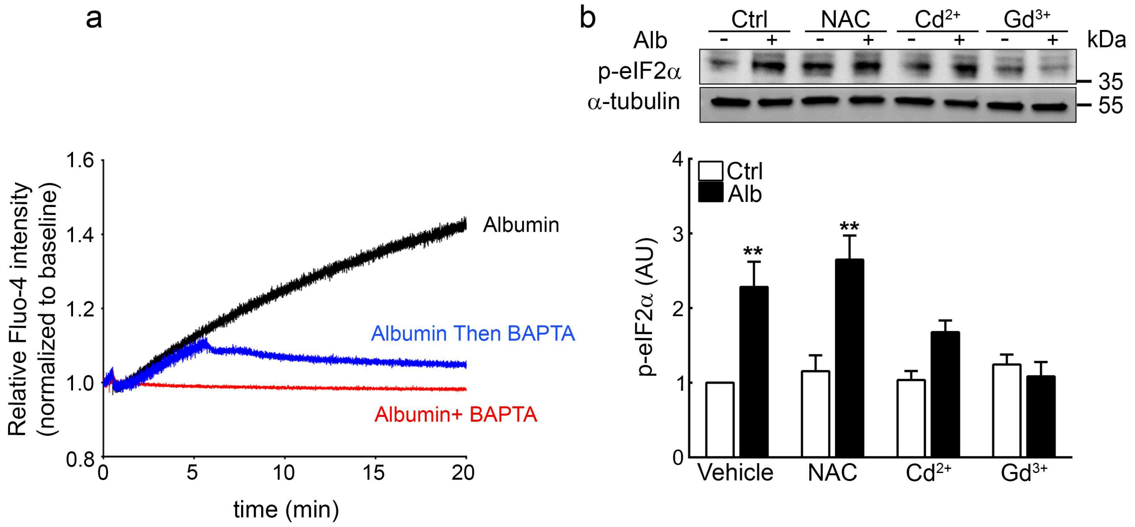

Supplementary Figure 2: Effect of extracellular calcium chelation, calcium entry inhibition and ROS inhibition during albumin exposure. (**a**) Measurement of intracellular calcium in mIMCD-3 cells exposed to 1% albumin for indicated times in control conditions (black trace), with Bapta before albumin exposure (Bapta, red trace) or 5min after albumin exposure (Albumin then Bapta, blue trace) (n = 3). **(b)** Representative blot (upper panel) and quantification (lower panel) of p-eIF2α evaluated in mIMCD-3 cells exposed to 1% albumin for 30 min in the presence or the absence of N-Acetyl Cysteine (NAC), Cd^{2+} or Gd^{3+} (n = 3). Data are mean ± SEM. Statistical analysis: one way ANOVA followed by Tukey-Kramer test, $^{**}P < 0.01$

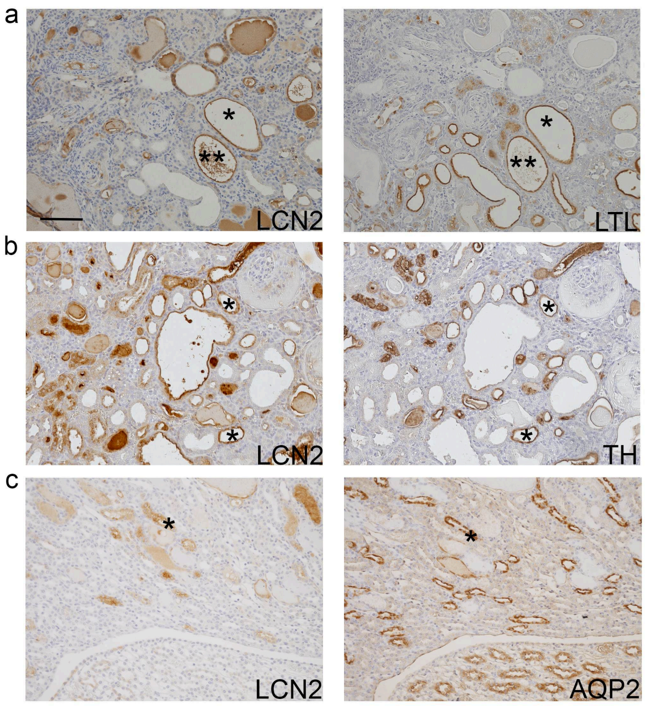

Supplementary Figure 3: LCN2 protein colocalizes with different tubular markers during proteinuria. Immunohistochemistry of LCN2 and **(a)** Lotus Tetraglobulin Lectin (LTL), a marker of proximal tubular cells, **(b)** Tamm-Horsfall protein (TH), a marker of Henle's loop cells, **(c)** and Aquaporin 2 (AQP2), a marker of collecting duct cells, performed on kidneys of 6-week-old $WT1^{+/mut}$ mice. Panels are representative samples of n = 3 per group, scale bar 100 μm.

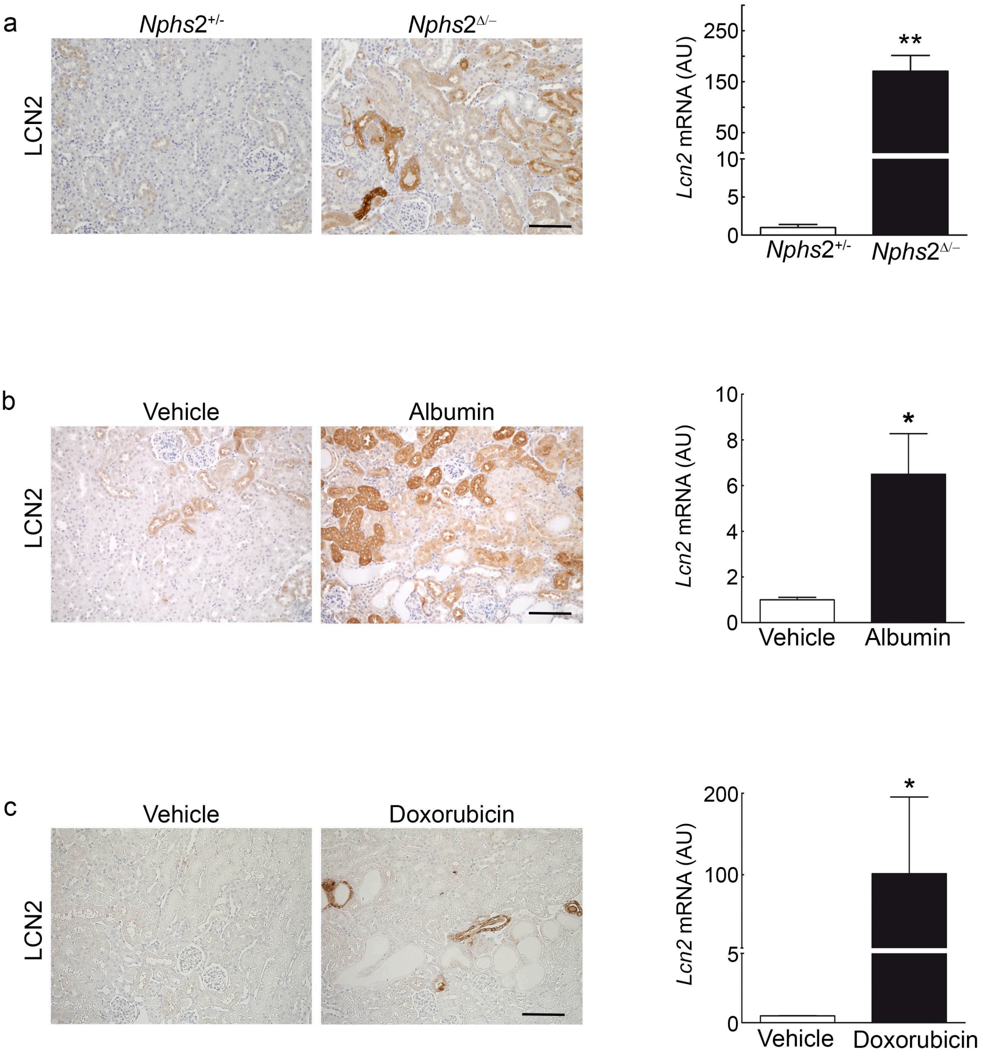

Supplementary Figure 4: Tubular LCN2 expression in different models of proteinuria. LCN2 protein (left panels) and mRNA (right panels) expression in **(a)** *Nphs2*$^{\Delta/-}$ mice (n = 6) and their control littermates (n = 3), **(b)** albumin (n = 4) and vehicle-injected (n = 3) mice, and **(c)** doxorubicin (n = 5) and vehicle-injected (n = 3) mice. Data are mean ± SEM. Statistical analysis: one way ANOVA followed by Tukey-Kramer test, $*P < 0.05$, $**P < 0.01$ *vs* *Nphs2*$^{+/-}$ or vehicle. Scale bar 100 μm.

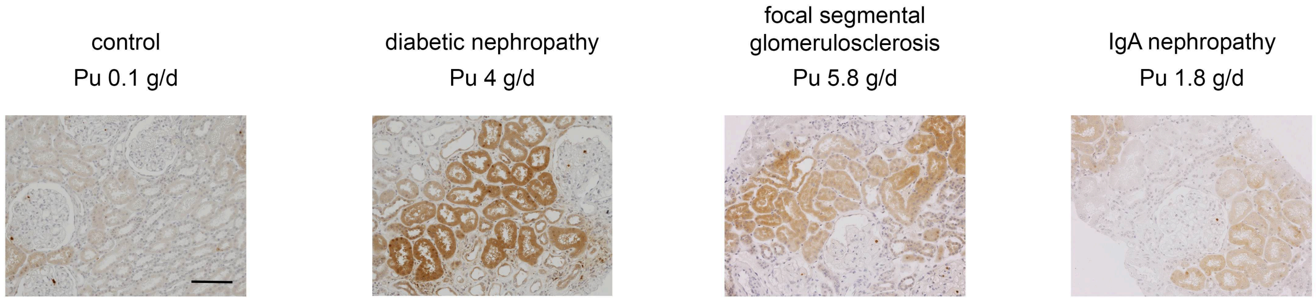

Supplementary Figure 5: LCN2 expression in human proteinuric kidney diseases. LCN2 protein expression in kidney biopsies of adult patients with significant proteinuria (Pu, evaluated in grams per day) from different etiologies (diabetic nephropathy, focal segmental glomerulosclerosis, IgA nephropathy). Panels are representative samples of n = 3 per group, scale bar 100 μm.

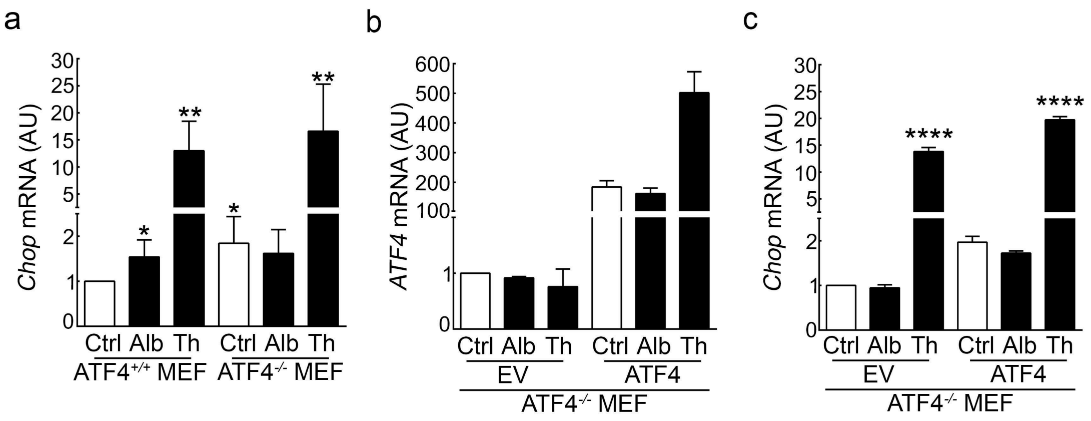

Supplementary Figure 6: CHOP expression in ATF4$^{-/-}$ cells upon ER stress induction and effect of ATF4 rescue. (a) CHOP mRNA expression in wild type *ATF4*$^{+/+}$ and *ATF4*$^{-/-}$ mouse embryonic fibroblasts (MEF) exposed to 1% albumin or 0.5 μM thapsigargin (Th) for 24h (n = 3). (b) ATF4 and (c) CHOP mRNA expression in *ATF4*$^{-/-}$ mouse embryonic fibroblasts (MEF) transfected with empty vector (EV) or ATF4 construct and exposed to 1% albumin or 0.5 μM thapsigargin (Th) for 24h (n = 3). Data are mean ± SEM. Statistical analysis: one way ANOVA followed by Tukey-Kramer test, * $P < 0.05$, ** $P < 0.01$, **** $P < 0.0001$ *vs* controls.

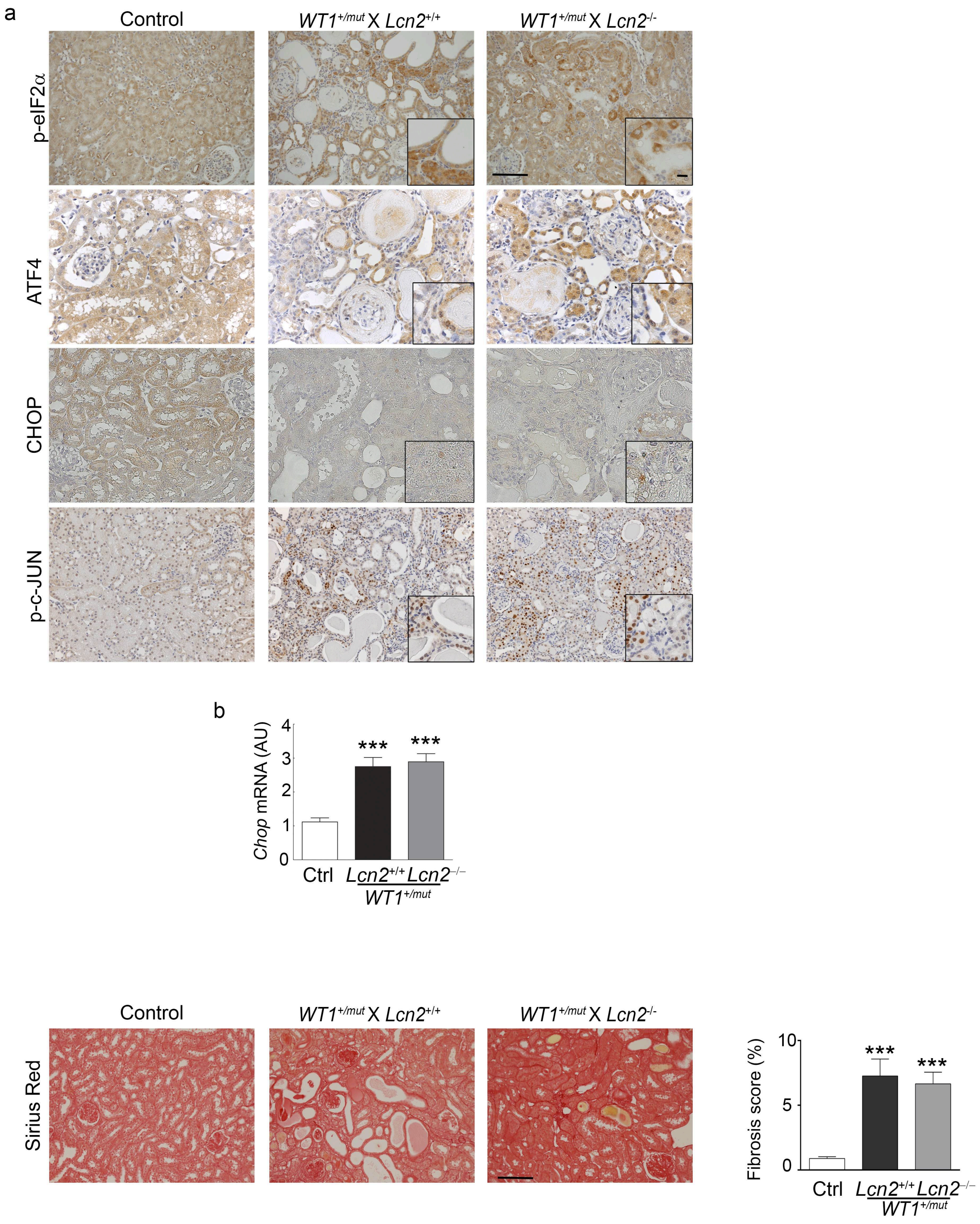

Supplementary Figure 7: Tubular cell UPR and fibrosis are not modulated by *Lcn2* inactivation in proteinuric mice. (a) p-eIF2a, ATF4, CHOP, and p-c-JUN expression (n = 5 per group, X200) and **(b)** CHOP mRNA expression in kidneys from controls (n = 7), $WT1^{+/mut}XLcn2^{+/+}$ (n = 10) and $WT1^{+/mut}XLcn2^{-/-}$ (n = 12) mice 6 weeks after birth. **(c)** Renal fibrosis (sirius red, left panel, X200) and quantification (right panel) in control (n = 6), $WT1^{+/mut}XLcn2^{+/+}$ (n = 6) and $WT1^{+/mut}XLcn2^{-/-}$ (n = 6) mice, 6 weeks after birth. Data are mean ± SEM. Statistical analysis: one way ANOVA followed by Tukey-Kramer test, ****P* < 0.01 *vs* controls. Scale bar 100 μm, insert scale bar 10 μm.

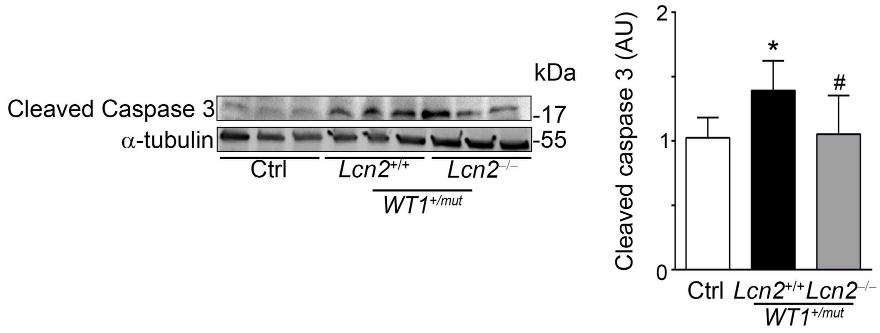

Supplementary Figure 8: Lcn2 deficiency protects from kidney cells apoptosis. Cleaved-caspase 3 protein expression in whole kidney extracts of control (n = 6), $WT1^{+/mut}XLcn2^{+/+}$ (n = 6) and $WT1^{+/mut}XLcn2^{-/-}$ (n = 6) mice, 6 weeks after birth (representative western blot left panel, quantification right panel). Data are mean ± SEM. Statistical analysis: one way ANOVA followed by Tukey-Kramer test, **P* < 0.05 *vs* controls, #*P* < 0.05 *vs* $WT1^{+/mut}XLcn2^{+/+}$.

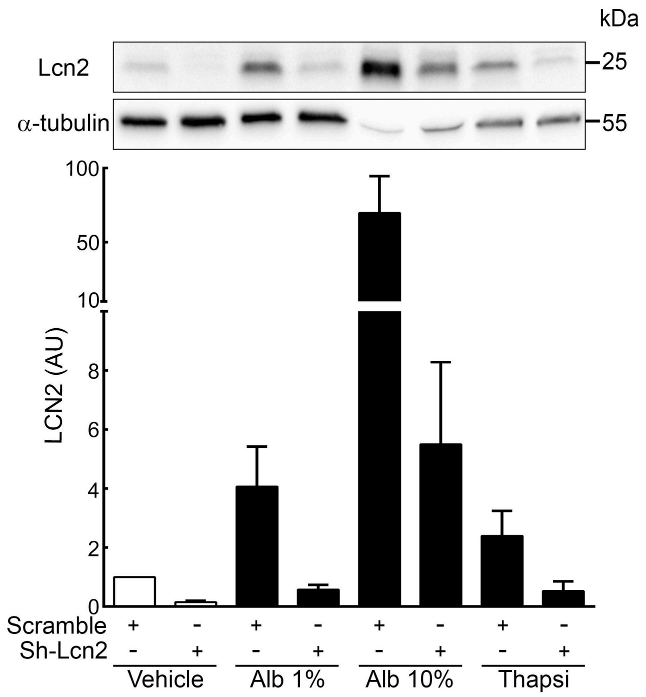

Supplementary Figure 9: Invalidation of Lcn2 by sh-RNA in m-IMCD3 cells. Lcn2 protein expression in scramble and Lcn2 sh-RNA expressing mIMCD-3 cells exposed to albumin (1% or 10% final) or thapsigargin for 24h (n = 3). Upper panel: representative western blot; lower panel: quantification. Data are mean ± SEM.

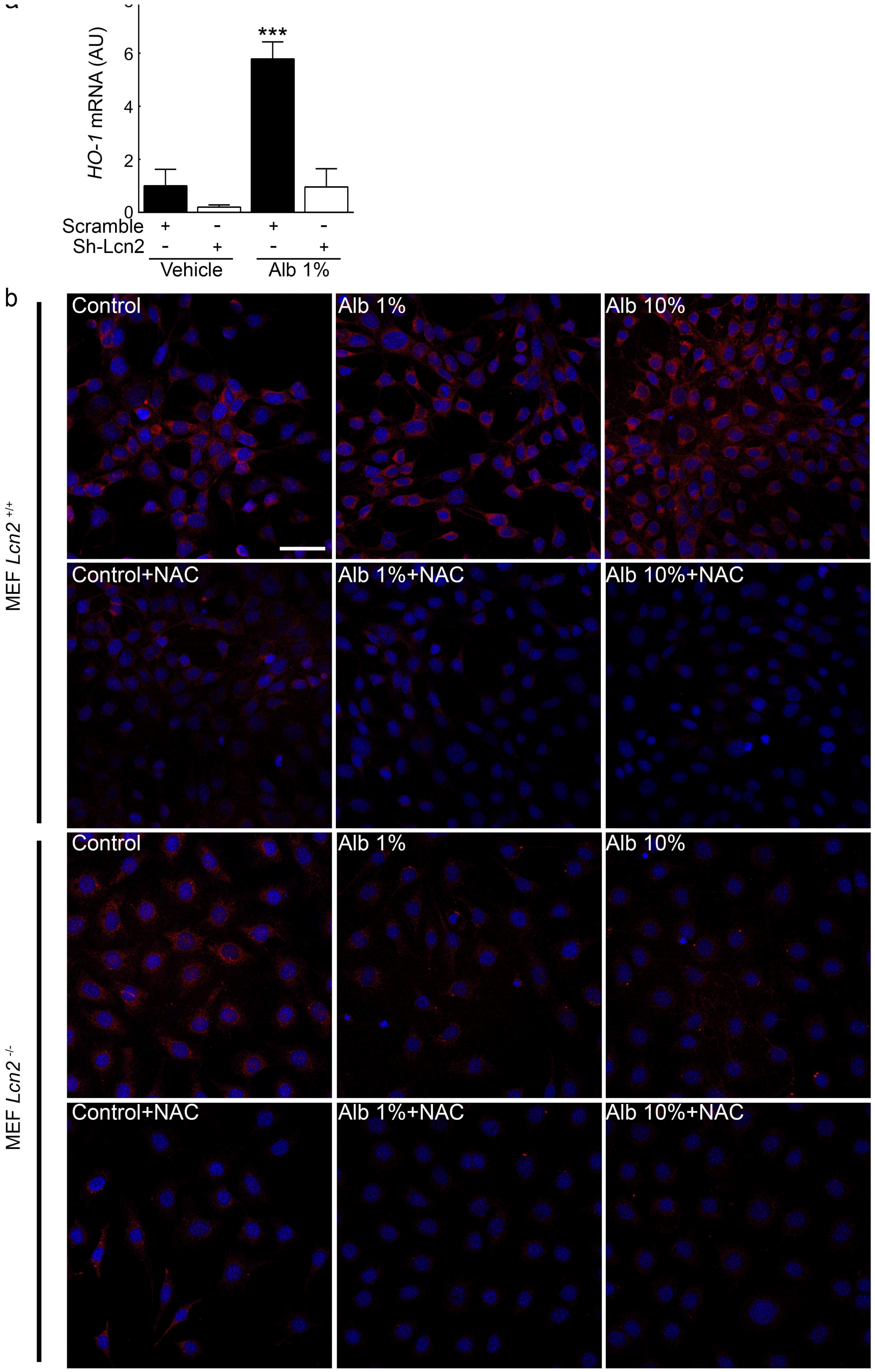

Supplementary Figure 10: Effect of Lcn2 deficiency and NAC treatment on intracellular ROS generation induced by albumin. **(a)** *Heme oxygenase 1 (HO-1)* mRNA expression in scramble and Lcn2 sh-RNA expressing mIMCD-3 cells exposed to albumin (1%) (n = 3). **(b)** Reactive oxygen species detection by Cell-ROX detection in fluorescent microscopy in MEF wild type or $Lcn2^{-/-}$ cells. Cells were treated with albumin 1% or 10% for 24h in the absence or the presence of NAC (n = 3). Data are mean ± SEM. Statistical analysis: one way ANOVA followed by Tukey-Kramer test, ****P* < 0.01 *vs* scramble vehicle. Scale bar 20 μm.

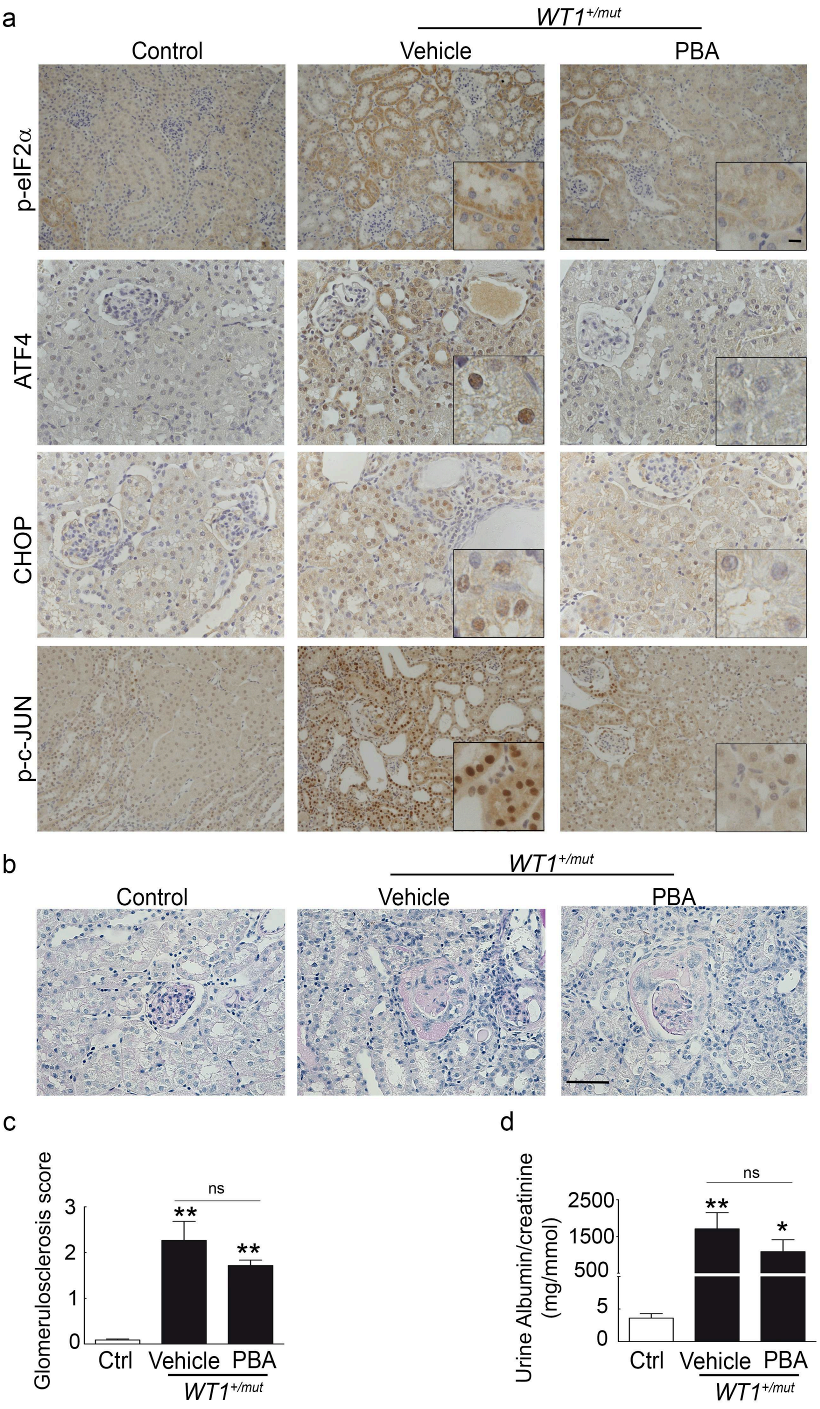

Supplementary Figure 11: PBA treatment inhibits tubular cell UPR, but does not modulate glomerular lesions and proteinuria in proteinuric mice. *WT1*$^{+/mut}$ mice were treated with 4-phenylbutyric acid (PBA) or vehicle for 2 weeks. Because no significant difference between vehicle and PBA-treated *WT1*$^{+/+}$ mice was observed, only one group (Control) is represented. **(a)** p-eIF2a, ATF4, CHOP, and p-c-JUN expression (n = 5 per group). **(b-c)** **(b)** Glomerular morphology (PAS staining), and **(c)** quantification of glomerular lesions (n = 6, n = 6 and n = 11 in control, vehicle-treated *WT1*$^{+/mut}$ and PBA-treated *WT1*$^{+/mut}$ groups, respectively). **(d)** Quantification of urine albumin/creatinine ratio. (n = 8, n = 5 and n = 11 in control, vehicle-treated *WT1*$^{+/mut}$ and PBA-treated *WT1*$^{+/mut}$ groups, respectively). Data are mean ± SEM. Statistical analysis: one way ANOVA followed by Tukey-Kramer test, *P < 0.05, **P < 0.05 *vs* controls**.** Scale bar 100 μm, insert scale bar 10 μm.

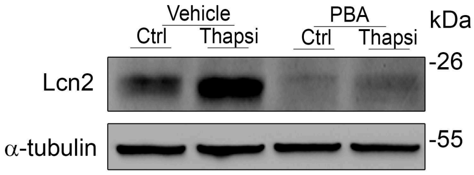

Supplementary Figure 12: PBA treatment inhibits thapsigargin-induced LCN2 expression in tubular cells. m-IMCD3 cells were exposed to thapsigargin 0.5 μM for 24h in the presence of either PBA or the vehicle. Representative western blot (n = 2).

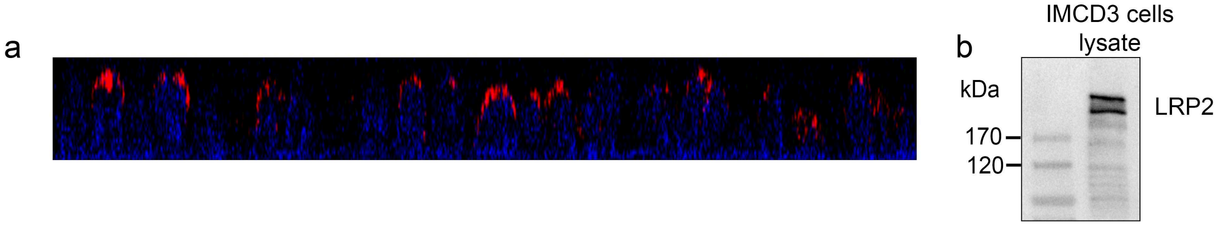

Supplementary Figure 13: m-IMCD3 cells express LRP2. **(a)** Immunofluorescence of LRP2 (red) and nuclear staining (blue) in m-IMCD3 cells. LRP2 protein is expressed at the apical membrane of the cells. **(b)** Western blot of LRP2 on m-IMCD3 cells lysate showing the expected molecular weight for LRP2 (517 kDa).

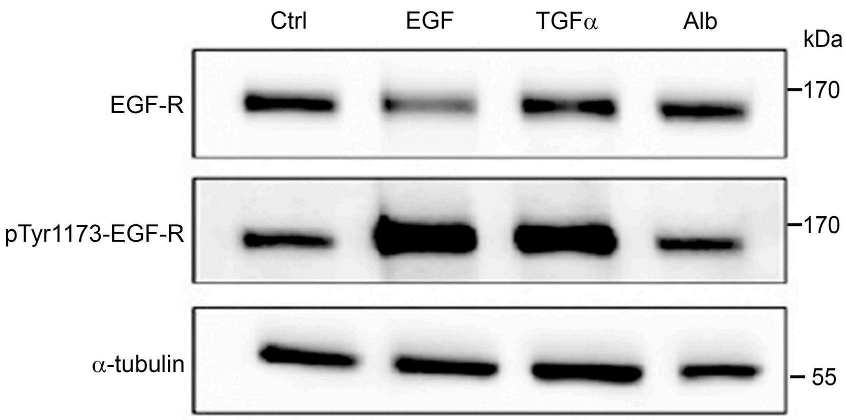

Supplementary Figure 14: Albumin exposure does not activate EGF-R phosphorylation. m-IMCD3 cells were exposed to EGF (10 nM), TGFα (10 nM) or Albumin (1%) for 10 min. Representative western blot for EGF-R and activated form of EGF-R (pTyr1173-EGF-R). Representative western blot (n = 3).

Figure 1.c pERK

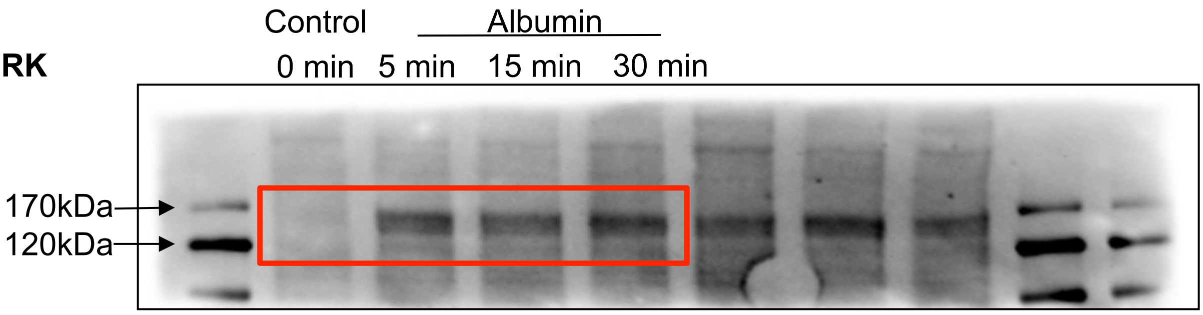

Figure 1.c peIF2α

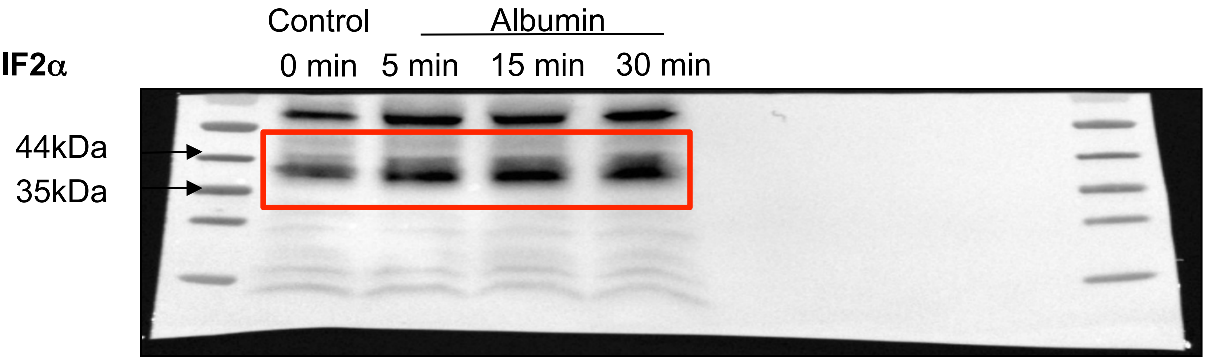

Figure 1.c p-c-Jun

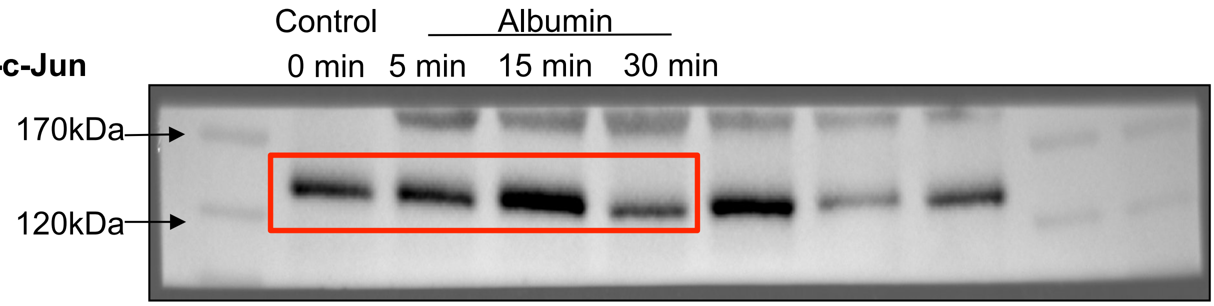

Figure 1.c α-Tubulin

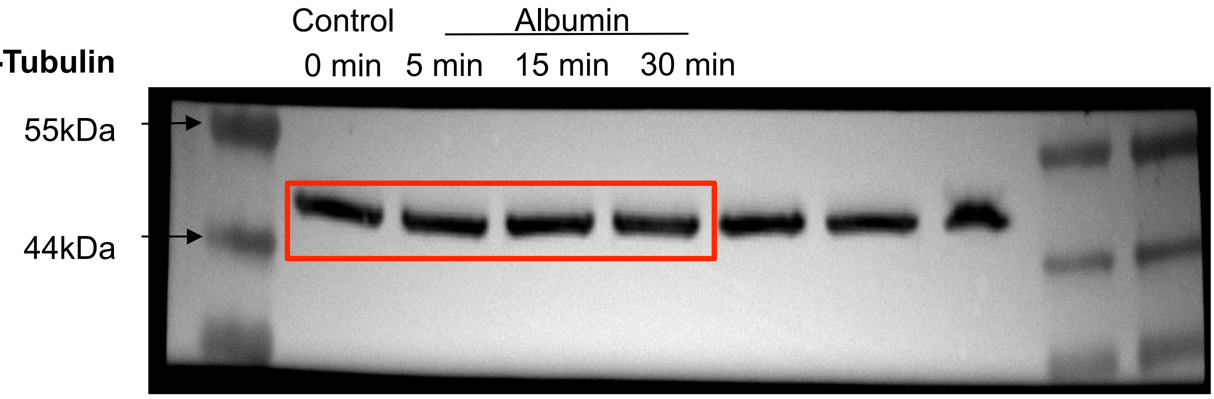

Supplementary Figure 15

Figure 1.d ATF4

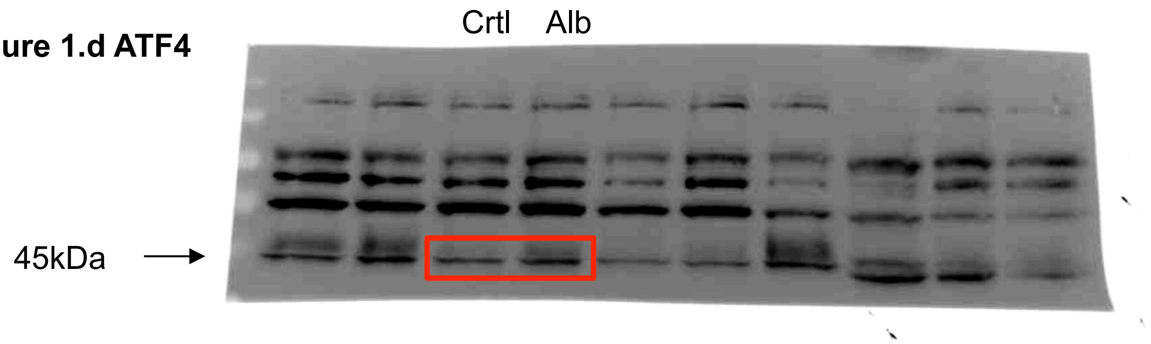

Figure 1.d CHOP

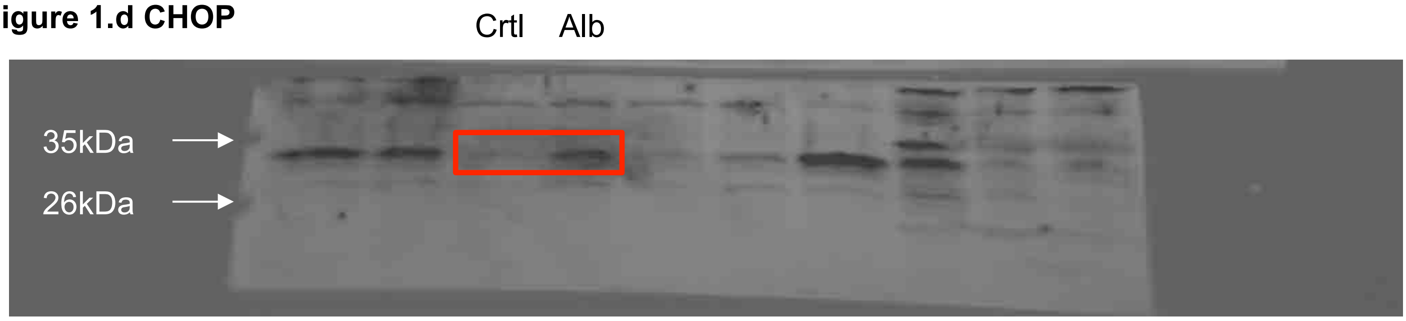

Figure 1.d α-Tubulin

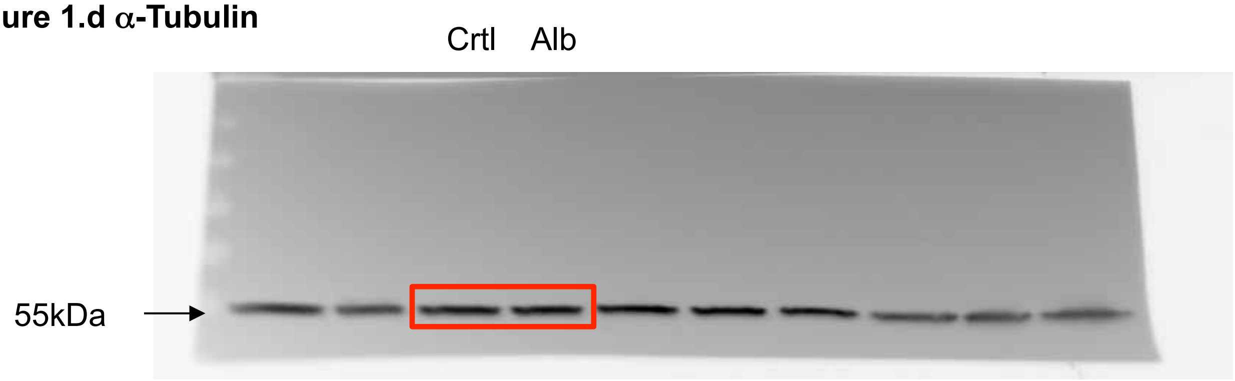

Supplementary Figure 15 continued

Figure 1.i pPERK

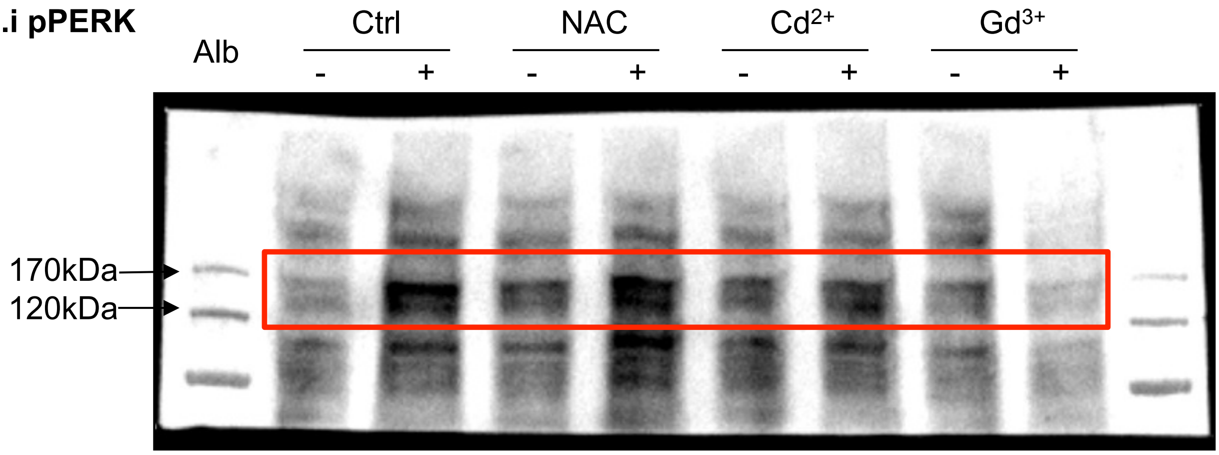

Figure 1.i α-Tubulin

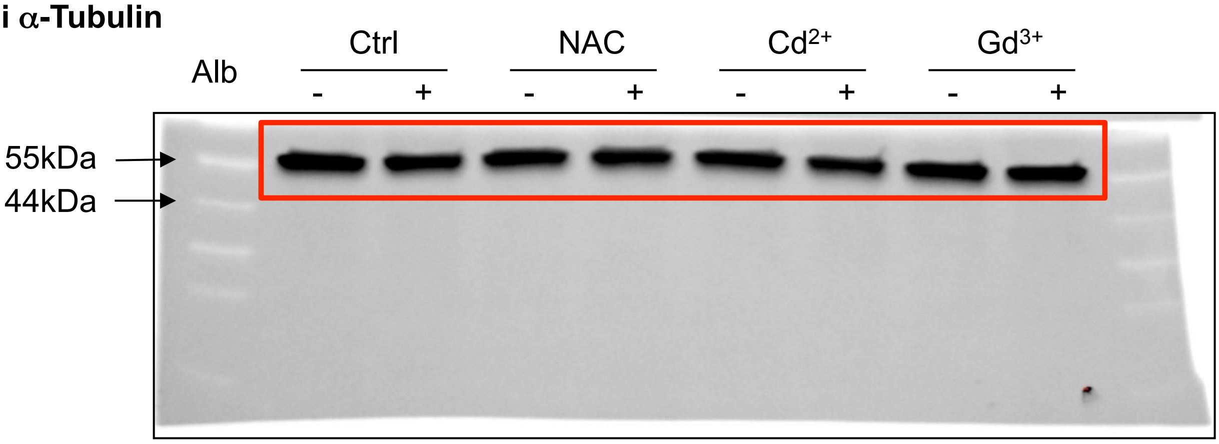

Supplementary Figure 15 continued

Figure 2.e Lcn2

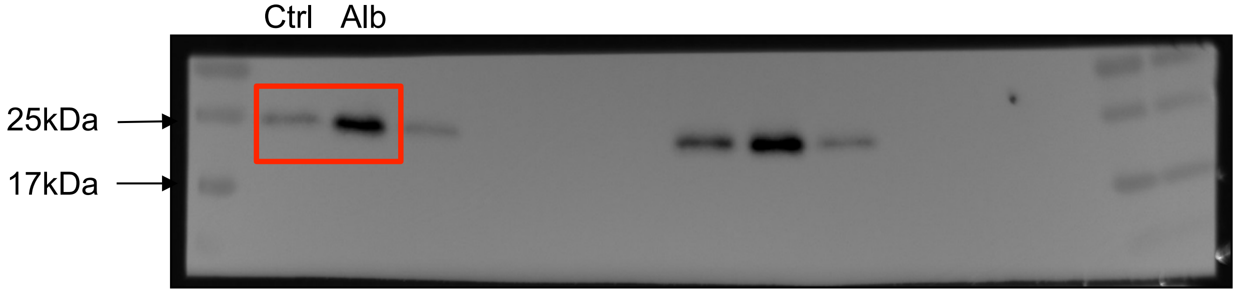

Figure 2.e α-Tubulin

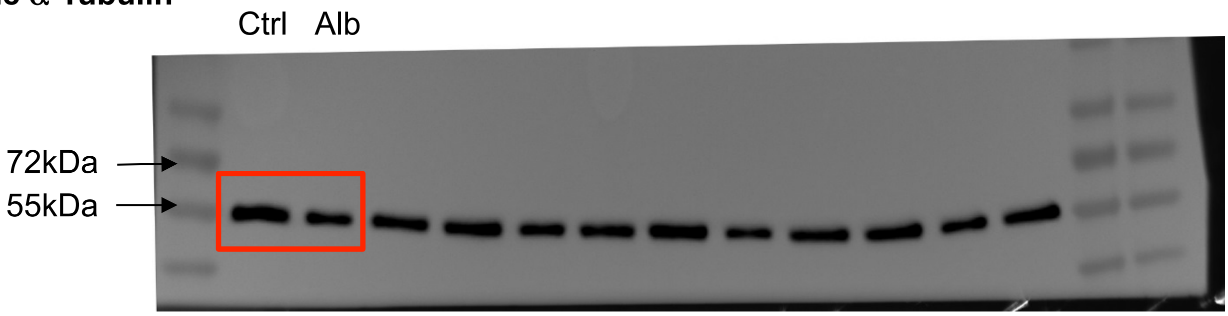

Figure 2.f Lcn2

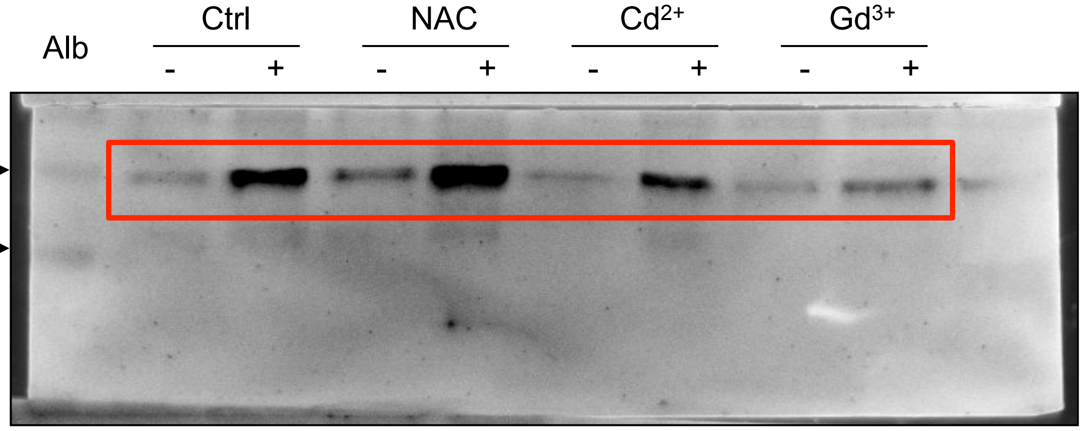

Figure 2.f α-Tubulin

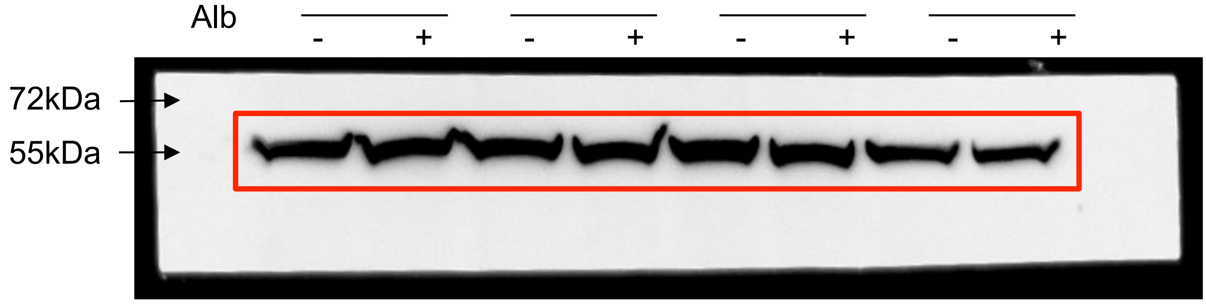

Supplementary Figure 15 continued

Figure 2.g Lcn2

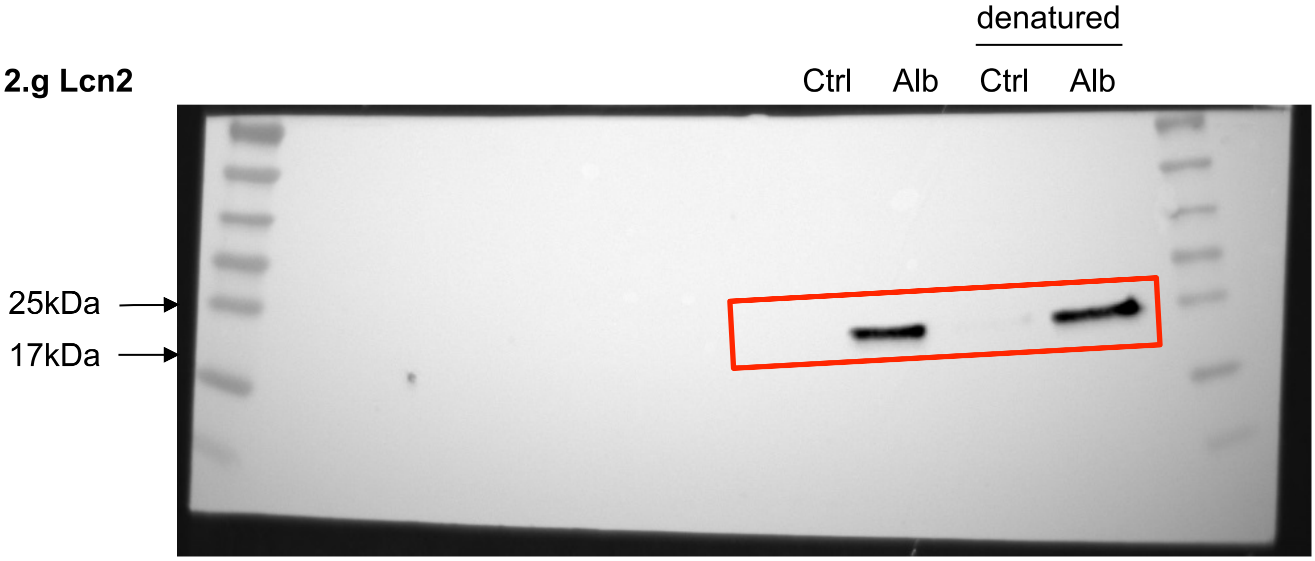

Figure 2.g α-Tubulin

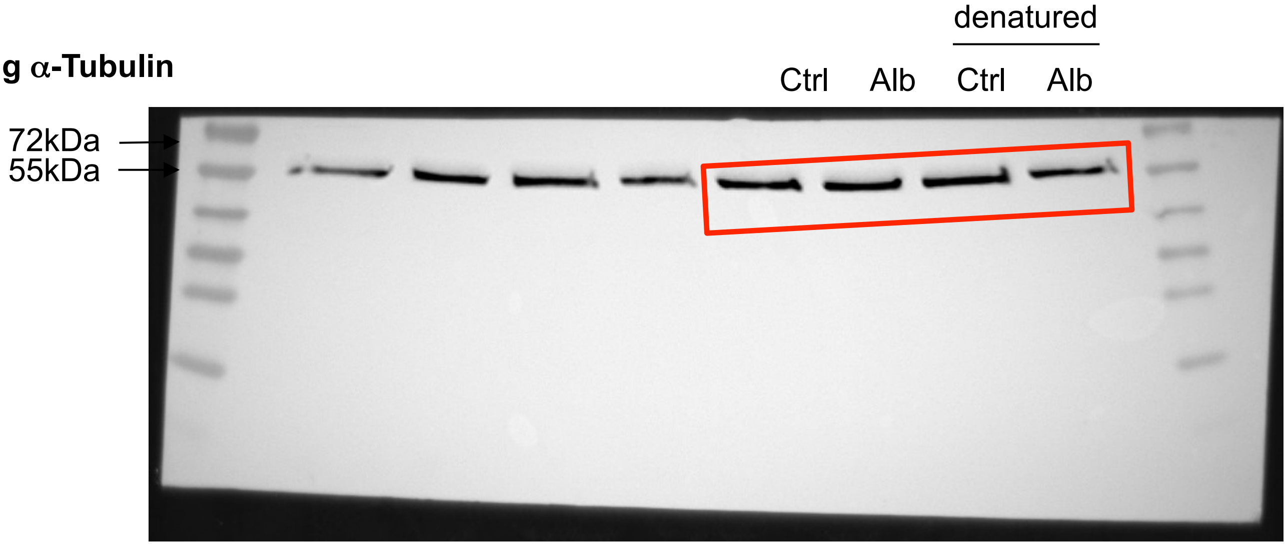

Figure 2.k Lcn2

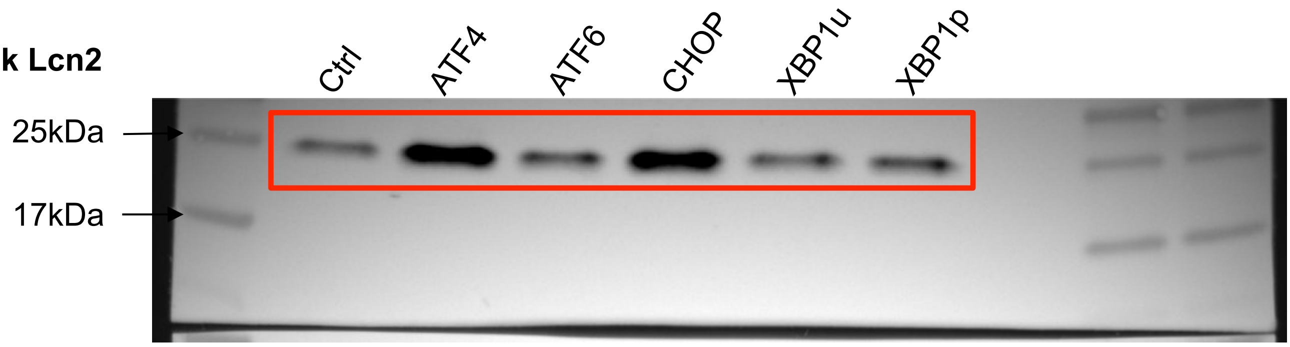

Figure 2.k α-Tubulin

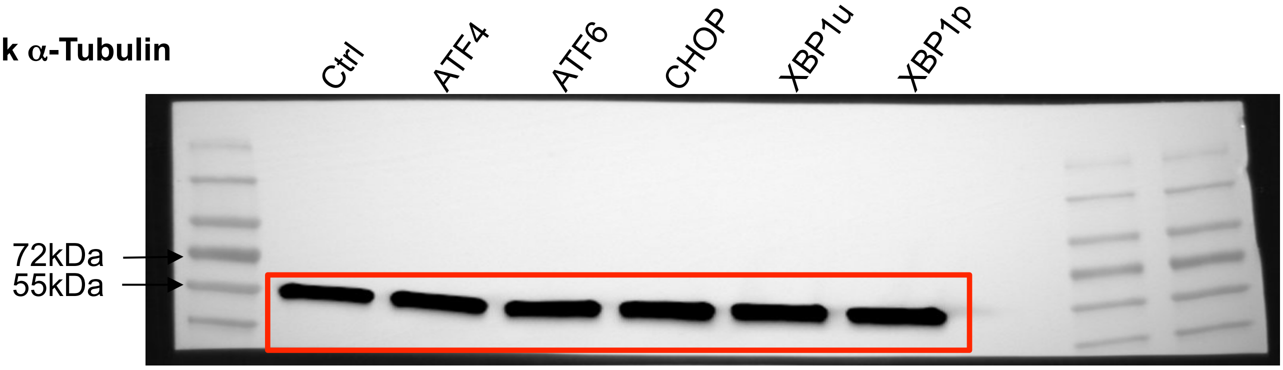

Supplementary Figure 15 continued

Figure 2.m Lcn2

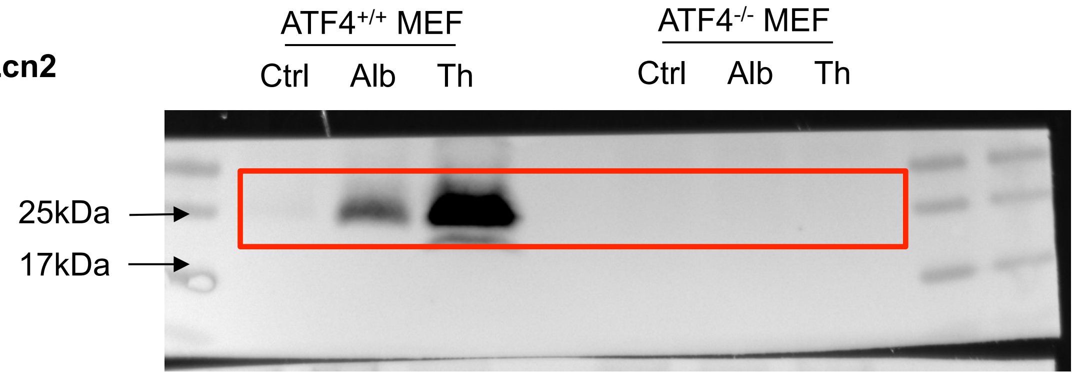

Figure 2.m α-Tubulin

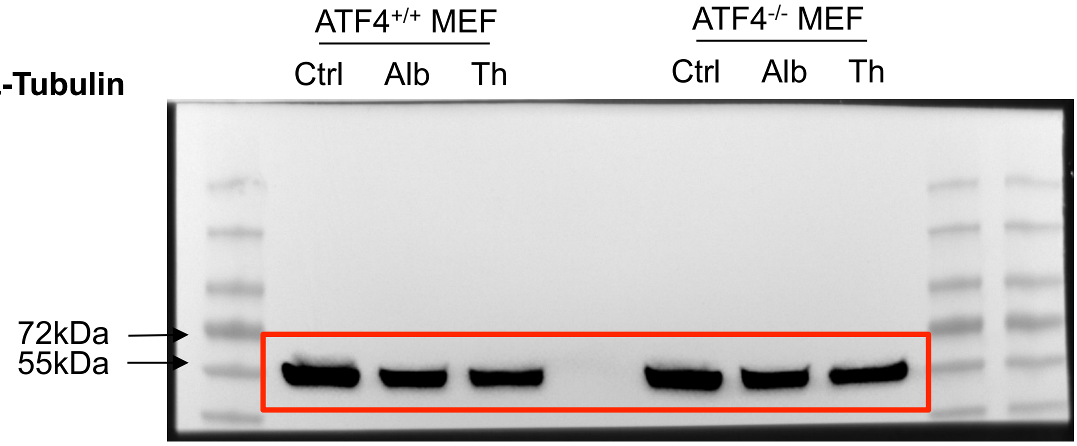

Figure 2.j Lcn2

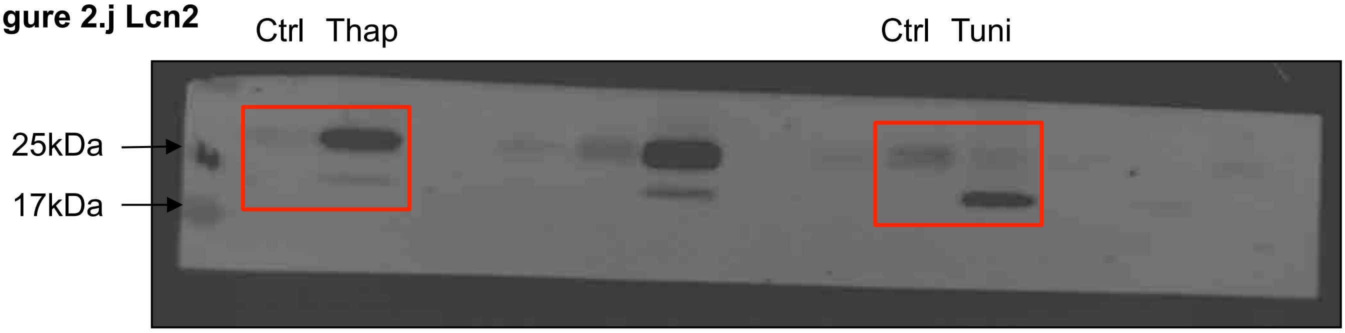

Figure 2.j α-Tubulin

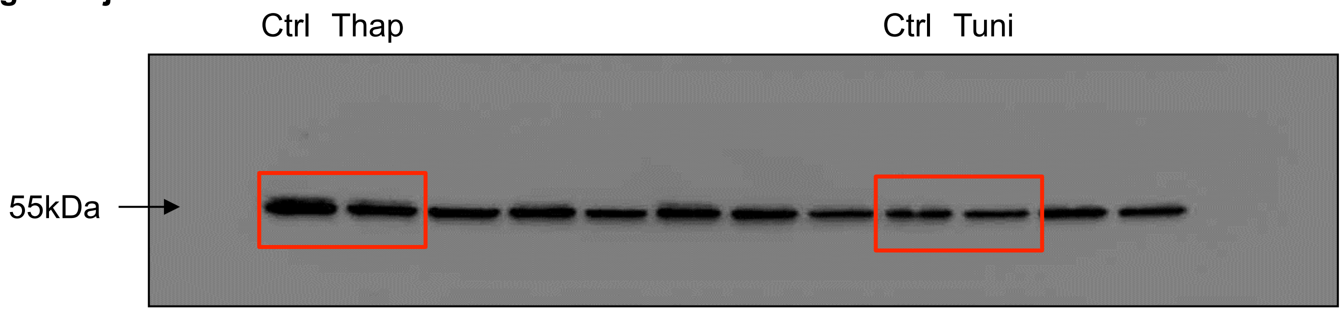

Supplementary Figure 15 continued

Figure 4.g ATF4

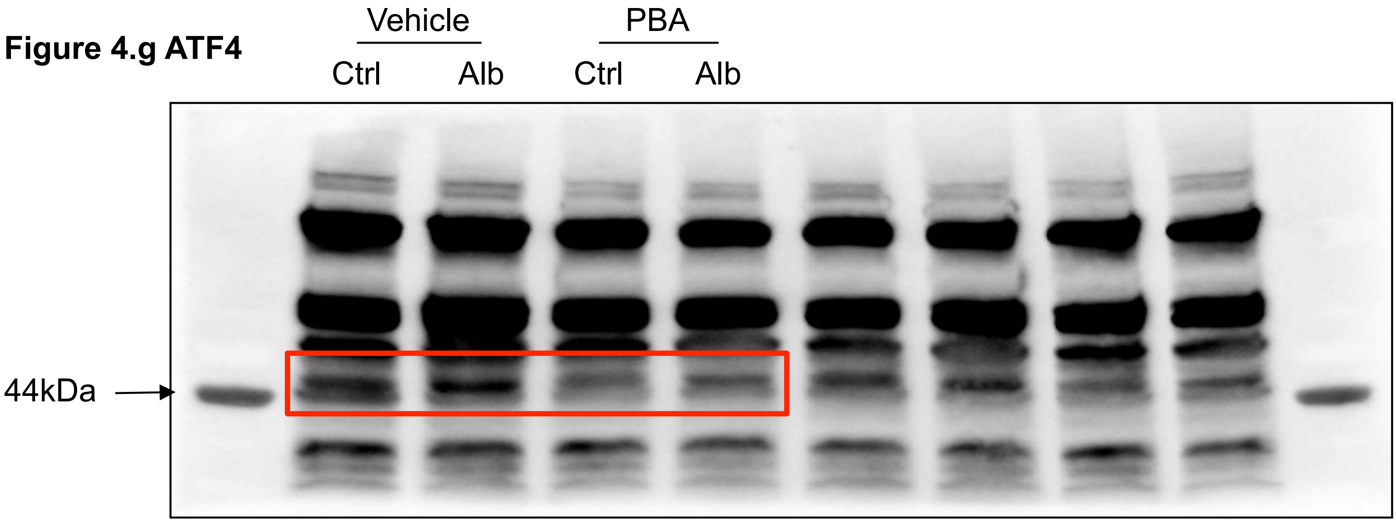

Figure 4.g Lcn2

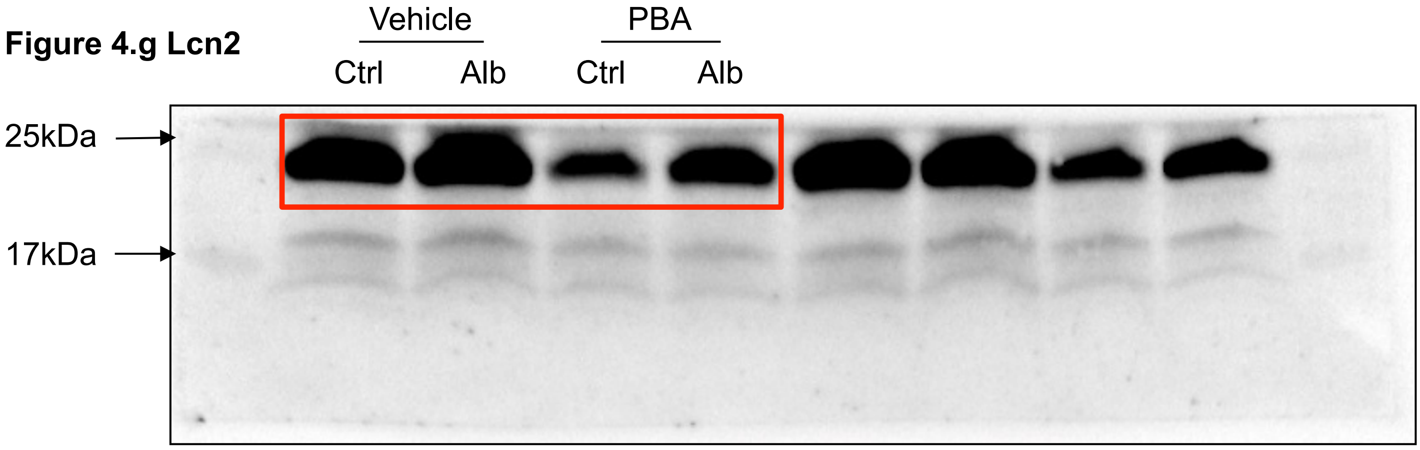

Supplementary Figure 15 continued

Supplementary Figure 2.b p-eIF2α

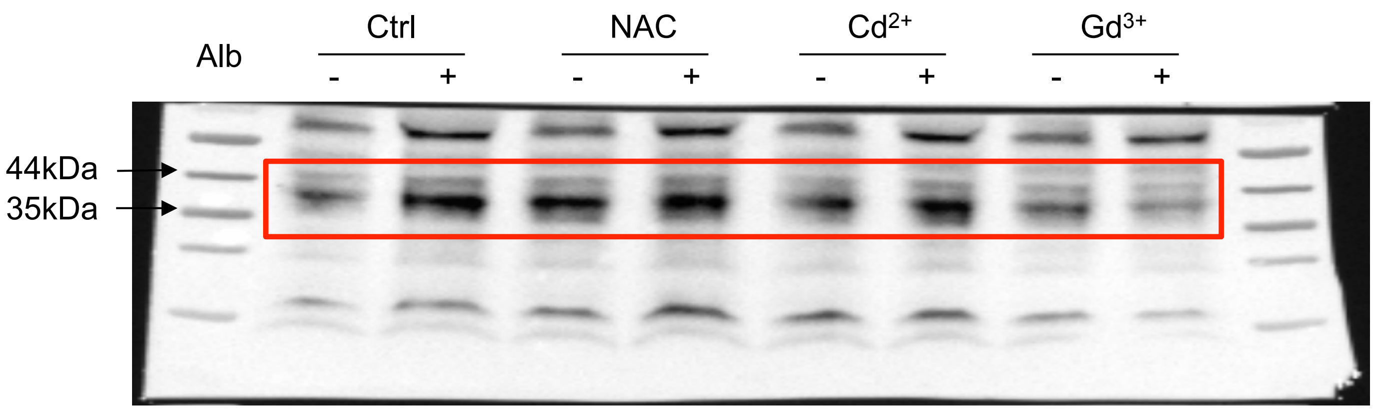

Supplementary Figure 8 Cleaved Caspase 3

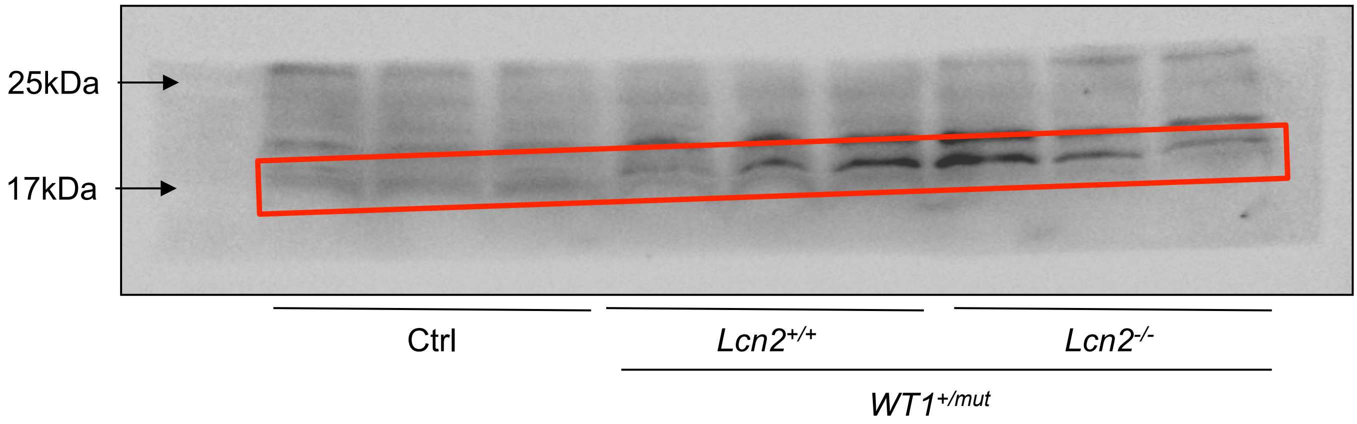

Supplementary Figure 8 α-Tubuline

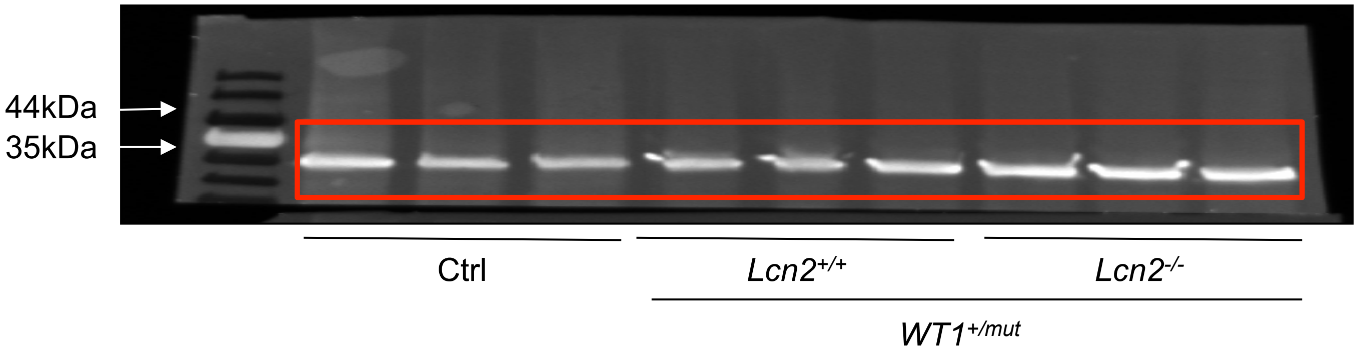

Supplementary Figure 15 continued

Supplementary Figure 9

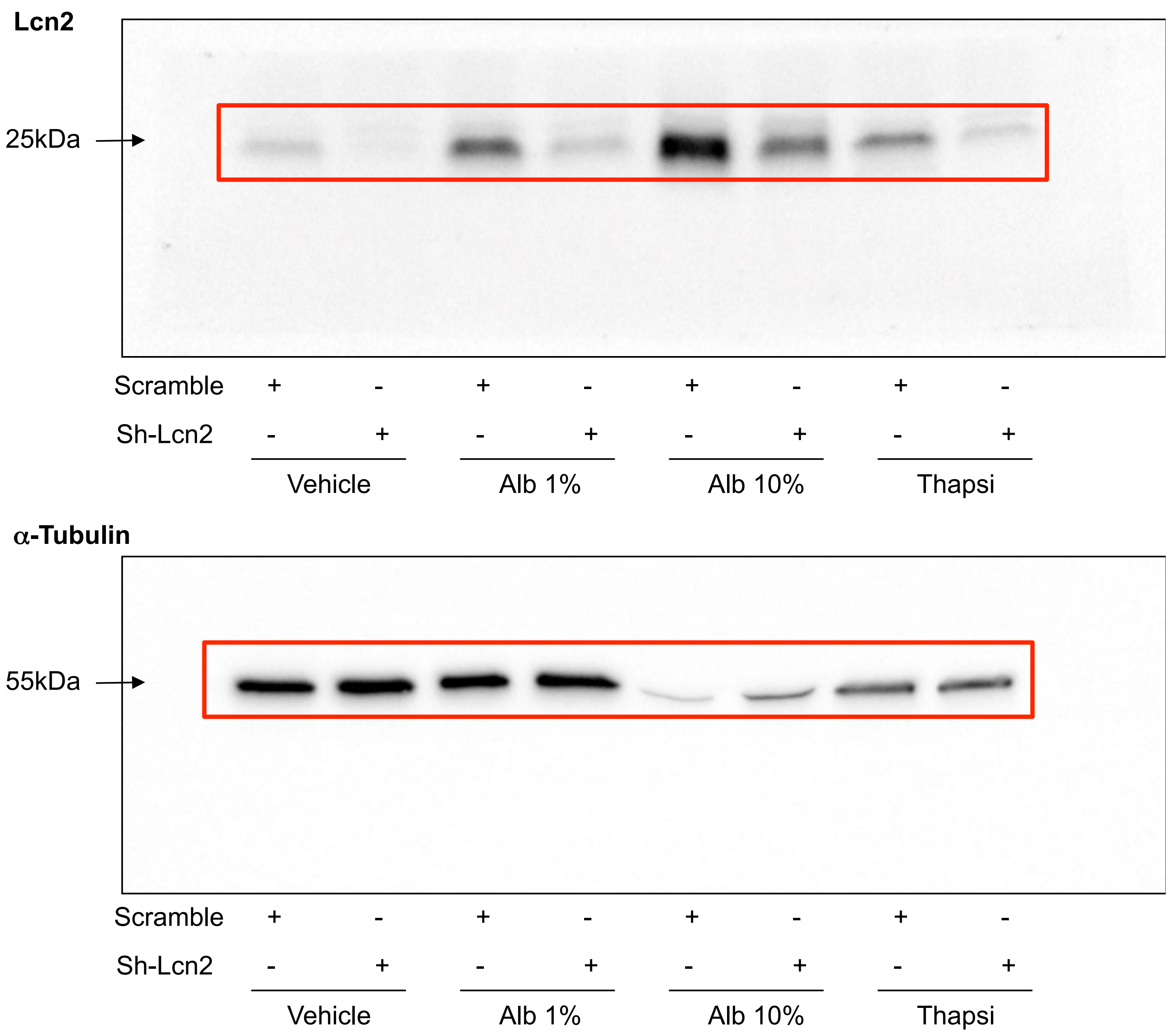

Supplementary Figure 15 continued

Supplementary Figure 12

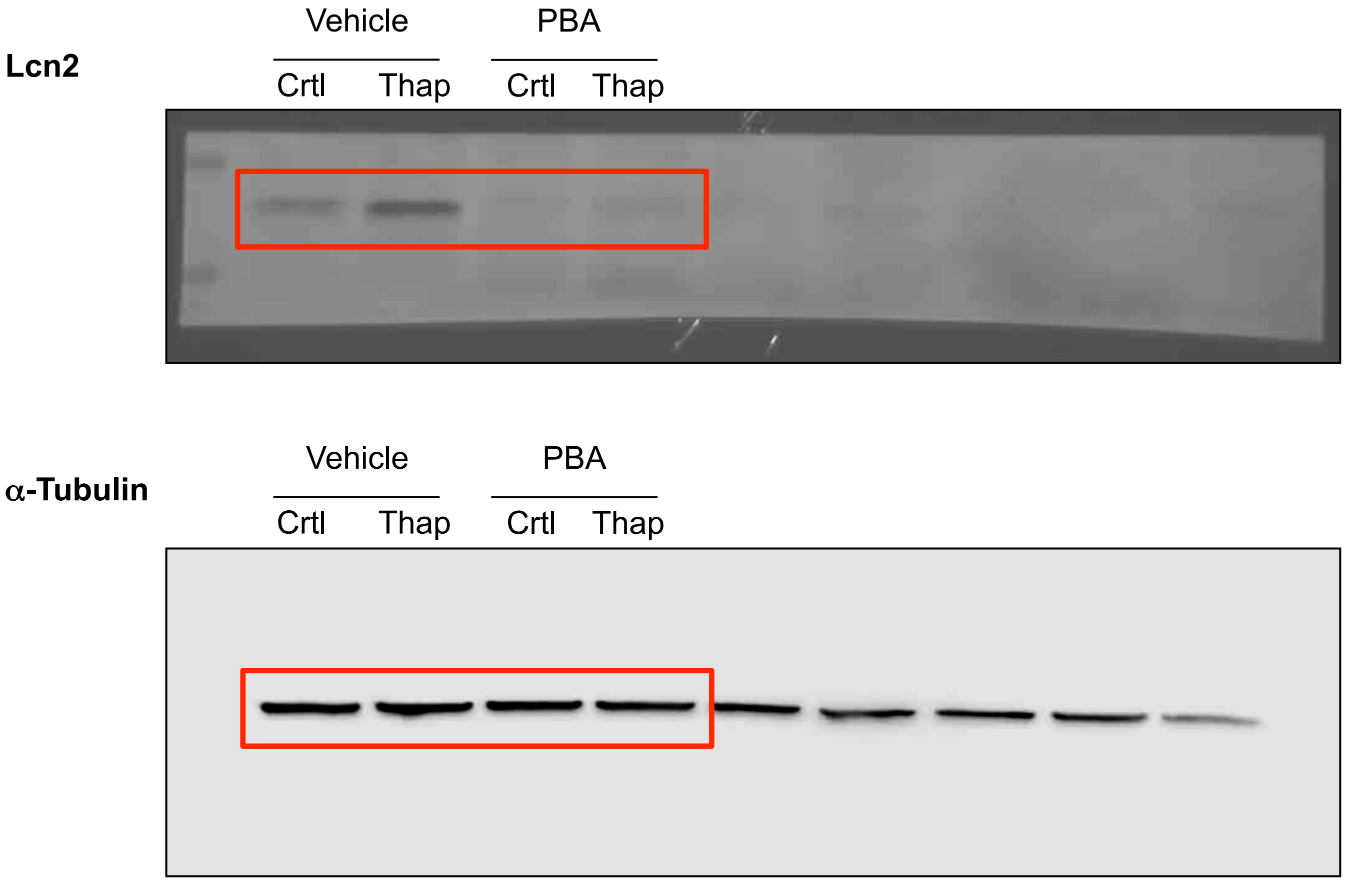

Supplementary Figure 15 continued
